# The association between circulating CD34+CD133+ endothelial progenitor cells and reduced risk of Alzheimer’s disease in the Framingham Heart Study

**DOI:** 10.37349/emed.2024.00216

**Published:** 2024-04-12

**Authors:** Yixuan Wang, Jinghan Huang, Ting Fang Alvin Ang, Yibo Zhu, Qiushan Tao, Jesse Mez, Michael Alosco, Gerald V. Denis, Anna Belkina, Ashita Gurnani, Mark Ross, Bin Gong, Jingyan Han, Kathryn L. Lunetta, Thor D. Stein, Rhoda Au, Lindsay A. Farrer, Xiaoling Zhang, Wei Qiao Qiu

**Affiliations:** 1 Biomedical Genetics, Department of Medicine, Boston University Chobanian & Avedisian School of Medicine, Boston, MA 02118, USA; 2 Department of Chemical Pathology, Faculty of Medicine, The Chinese University of Hong Kong, Hong Kong, China; 3 Department of Anatomy & Neurobiology, Boston University Chobanian & Avedisian School of Medicine, Boston, MA 02118, USA; 4 Alzheimer’s Disease Research Center, Boston University Chobanian & Avedisian School of Medicine, Boston, MA 02118, USA; 5 Department of Pharmacology, Physiology and Biophysics, Boston University Chobanian & Avedisian School of Medicine, Boston, MA 02118, USA; 6 Department of Neurology, Boston University Chobanian & Avedisian School of Medicine, Boston, MA 02118, USA; 7 Framingham Heart Study, Boston University Chobanian & Avedisian School of Medicine, Boston, MA 02118, USA; 8 Hematology & Medical Oncology, Department of Medicine, Boston University Chobanian & Avedisian School of Medicine, Boston, MA 02118, USA; 9 Department of Pathology & Laboratory Medicine, Boston University Chobanian & Avedisian School of Medicine, Boston, MA 02118, USA; 10 School of Energy, Geosciences, Infrastructure and Society, Institute of Life and Earth Sciences, Heriot-Watt University, EH14 4AS Edinburgh, UK; 11 Department of Pathology, University of Texas Medical Branch, Galveston, TX 77555, USA; 12 Vascular Biology, Department of Medicine, Boston University Chobanian & Avedisian School of Medicine, Boston, MA 02118, USA; 13 Departments of Biostatistics, Boston University School of Public Health, Boston, MA 02118, USA; 14 VA Boston Healthcare System, Boston, MA 02132, USA; 15 Departments of Epidemiology, Boston University School of Public Health, Boston, MA 02118, USA; 16 Department of Ophthalmology, Boston University Chobanian & Avedisian School of Medicine, Boston, MA 02118, USA; 17 Department of Psychiatry, Boston University Chobanian & Avedisian School of Medicine, Boston, MA 02118, USA

**Keywords:** Alzheimer’s disease, CD34+CD133+, circulating endothelial progenitor cells, vascular diseases, genome-wide association studies (GWAS)

## Abstract

**Aim:**

Endothelial dysfunction has been associated with both cerebrovascular pathology and Alzheimer’s disease (AD). However, the connection between circulating endothelial cells and the risk of AD remains uncertain. The objective was to leverage data from the Framingham Heart Study to investigate various circulating endothelial subtypes and their potential correlations with the risk of AD.

**Methods:**

The study conducted data analyses using Cox proportional hazard regression and linear regression methods. Additionally, genome-wide association study (GWAS) was carried out to further explore the data.

**Results:**

Among the eleven distinct circulating endothelial subtypes, only circulating endothelial progenitor cells (EPCs) expressing CD34+CD133+ were found to be negatively and dose-dependently associated with reduced AD risk. This association persisted even after adjusting for age, sex, years of education, apolipoprotein E (*APOE*) ε4 status, and various vascular diseases. Particularly noteworthy was the significant association observed in individuals with hypertension and cerebral microbleeds. Consistently, positive associations were identified between CD34+CD133+ EPCs and specific brain regions, such as higher proportions of circulating CD34+CD133+ cells correlating with increased volumes of white matter and the hippocampus. Additionally, a GWAS study unveiled that CD34+CD133+ cells influenced AD risk specifically in individuals with homozygous genotypes for variants in two stem cell-related genes: kirre like nephrin family adhesion molecule 3 (*KIRREL3*, rs580382 CC and rs4144611 TT) and exocyst complex component 6B (*EXOC6B*, rs61619102 CC).

**Conclusions:**

The findings suggest that circulating CD34+CD133+ EPCs possess a protective effect and may offer a new therapeutic avenue for AD, especially in individuals with vascular pathology and those carrying specific genotypes of *KIRREL3* and *EXOC6B* genes.

## Introduction

Endothelial pathology in the brain co-exists with neuropathological hallmarks of Alzheimer’s disease (AD) and is shown to increase the risk of late-onset AD [1-3]. Microvascular endothelium, which is composed of blood-facing cells of the blood-brain barrier (BBB) in brain capillary beds, may play an important role in the development of AD during peripheral chronic inflammation [1, 4, 5]. Our preclinical study demonstrates that monomeric C-reactive protein (mCRP) and apolipoprotein E (*APOE*) protein compete to bind with endothelial membrane surface CD31 protein in the brain during peripheral inflammation [6]. mCRP-CD31 binding causes shortened microvasculature in the brain, leading to AD pathology and cognitive impairment. As the major AD genetic factor, *APOE* ε4, leads to endothelial dysfunction, cerebrovascular pathology, and AD pathogenesis [6], it is unclear but important to examine if any subtype(s) of circulating endothelial cells would be associated with AD risk.

Blood circulating endothelial cells can be divided into two major types: 1) endothelial mature cells (EMCs) expressing CD31 and 2) endothelial progenitor cells (EPCs) with different subtypes expressing CD34, CD133, or both proteins. While peripheral chronic inflammation causes endothelial damage marked by CD31 expression [2, 7, 8], some studies have suggested that CD34+CD133+ EPCs are prognostic biomarkers [9] and can be therapeutic for ischemic diseases [10-12]. As both peripheral inflammation and vascular diseases are shown to increase AD risk, two cross-sectional studies showed that AD patients had a lower number of circulating CD34+CD133+ cells [13] or CD34+ cells [14] than controls. Thus, it is important to study if and which circulating endothelial subtypes are related to AD risk, especially in the presence of cerebrovascular pathology, in a longitudinal study.

In this study, we examined the relationship between different subtypes of circulating EPCs and EMCs, vascular pathologies, AD/dementia risk, and brain volumes in participants of the Framingham Heart Study (FHS) Offspring Generation 2 (Gen 2) cohort. These FHS participants have been longitudinally followed for AD dementia incidence. Among different circulating endothelial cells, we found that CD34+CD133+ EPCs were significantly associated with reduced AD risk, especially among those with vascular diseases. We further conducted a genome-wide association study (GWAS) to identify loci associated with AD risk through their interaction with CD34+CD133+ EPCs. Finally, we used the RNA-seq expression data from the Religious Orders Study/Memory and Aging Project (ROSMAP) study to explore the relationship between the expressions of the identified genes and AD.

## Materials and methods

### Participants

FHS is a single-site, multi-generation, community-based, prospective cohort study of health in Framingham, Massachusetts. The current study included 1,566 participants (Mean_age_ = 65.93 ± 8.91; 46.36% female) from the Offspring cohort (Gen 2) with data on genome wide single nucleotide polymorphisms (SNPs) and circulating CD34+CD133+ cells measured at examination 8 (2005–2007) who were followed subsequently for incident AD dementia. Relevant details about this cohort have been previously described [15]. Fifty-nine of them developed incident AD through the period ending in 2019 (Figure S1). Informed consent was obtained from all participants and the study protocol was approved by the Institutional Review Board of Boston University.

### Circulating EPCs count method

We used the existing data on circulating EPCs and EMCs in the FHS cohort, and the data has been published in several papers [16-18]. Briefly, fasting blood samples were collected from participants in order to count circulating progenitor cells and were stored at 2–8°C [19] and peripheral blood mononuclear cells (PBMCs) were used for cell phenotyping [17]. Then, PBMCs were isolated via Ficoll (General Electric) gradient centrifugation, counted, incubated with FcR blocking antibodies (Miltenyi Biotec), labeled with anti-human KDR PE antibody (R&D Systems), anti-human CD34 FITC antibody (BD Biosciences), anti-human CD133 APC antibody (BD Biosciences). Samples were washed and fixed with 2% paraformaldehyde. Cells were analyzed on BD FACSCalibur cytometer running CellQuest software (BD Biosciences). Data were analyzed using Flowjo 8 (Flowjo LLC) and positive populations were identified by comparisons of stained samples with samples incubated with isotype-matching IgG antibodies conjugated to FITC and PE. Compensation was performed using single-stain controls. Percentages of CD34+, CD34+CD133+, CD34+CD133−, CD34−CD133+, CD34−CD133−, CD34+, and CD34+KDR+(VEGFR2) cells were quantified in the data. Similarly, anti-human CD31 PE (BD Biosciences) and anti-human CD45 PerCP antibody (BD Biosciences) were used to analyze CD31+, CD31−, CD31+CD45−, CD31+DIM, and CD31+lymphoid cells. We used the data of both the proportions of each cell type among the total number of PBMCs (%) and the concentrations of each cell type (cell number per mL) shown in Table 1.

### Dementia/AD diagnoses

The laboratory researchers who conducted the measurements of different circulating cells were not involved in the process of AD dementia diagnoses and were blind for the cognitive status of the participants. All of the Gen 2 participants were followed for incident AD and dementia. The Mini-Mental State Examination (MMSE) was administered beginning at the time of the 5th health exam (i.e., 1991–1995) to monitor changes in cognitive status. A decrease in MMSE performance of 3 or more points from the immediately preceding exam or 5 or more points across all the exams would indicate a change in cognitive status that warranted review by a dementia diagnostic panel consisting of at least one neurologist and one neuropsychologist. Furthermore, from 1999–2005, all the surviving Gen 2 participants were invited for an in-depth cognitive examination, which also screened for incident cognitive impairment that warranted review by the dementia diagnostic panel. Consensus diagnostic procedures according to the criteria of Diagnostic and Statistical Manual of Mental Disorders (DSM-IV) and National Institute of Neurological and Communicative Diseases and Stroke/Alzheimer’s Disease and Related Disorders Association (NINCDS-ADRDA) for dementia and AD were conducted and have been previously described [20]. Incidences of dementia and AD after the CD34+CD133+ measurement at exam 8 were used for the analyses.

### Brain measurements

We used the existing data on brain volumes from FHS for this study. The brain magnetic resonance imaging (MRI) protocol has been reported in detail elsewhere [21, 22]. A Siemens 1-T MR machine with a T2-weighted double spin-echo coronal imaging sequence was used (Siemens Healthineers, Erlangen, Germany). A central laboratory blinded to demographic and clinical information processed digital information on brain images and quantified the brain data with a custom-written computer program operating on a UNIX, Oracle Solaris platform (Sun Microsystems, Santa Clara, California, USA). The semiautomated segmentation protocol for quantifying gray matter *vs.* white matter volumes in total cerebral brain volume (TCV), frontal lobar, parietal lobe, occipital lobe, and temporal lobe brain volume as well as hippocampal volume (HPV) has been described elsewhere [23], as has the interrater reliabilities for these methods. TCV was determined using a convolutional neural network method [15]. Non-linear co-registration of images to the Desikan-Killiany-Tourville atlas [16] enabled calculation of regional gray matter volumes [17, 18]. MRI volumes were corrected for head size by calculating the percentage of TCV. Each image set underwent rigorous quality control including assessments of the original acquisition and image processing quality.

### Peripheral vascular diseases and cerebrovascular diseases

#### Coronary heart disease

Participants were diagnosed as having developed coronary heart disease (CHD) if upon review of the case, a panel of three investigators (The Framingham Endpoint Review Committee) agreed on one of the following definite manifestations: myocardial infarction, coronary insufficiency, angina pectoris, sudden death from CHD, or non-sudden death from CHD.

#### Hypertension

We used treatment for hypertension (HTN) as a proxy for having HTN. Treatment for HTN was based on evidence of prescribed HTN medication use, self-report or FHS doctor’s report of treatment for HTN at the time of the exam. If a discrepancy between these three sources of information occurred, a chart review of medical records was conducted to make a final determination of HTN treatment.

#### Stroke

The diagnosis of stroke was based on the occurrence of a clinically evident stroke documented by clinical records that were reviewed by at least two neurologists. Stroke was defined as the sudden or rapid onset of a focal neurologic deficit persisting for greater than 24 hours.

#### Silent brain infarcts and cerebral microbleeds

MRI infarcts in persons without a clinical stroke documented by the Framingham Stroke surveillance team, were considered silent brain infarct [24] and cerebral microbleeds (CMB) were examined by visual inspection of brain MRI scans as have been previously described [25]. Exclusion criteria for the brain MRI scan included those who could not undergo MRI due to claustrophobia, presence of a pacemaker, or any other source of metal in their body.

#### White matter hyperintensity

White matter hyperintensity (WMHI) volumetric measures were extracted from a combination of FLAIR and 3D T1 images with a modified Bayesian probability structure based on a previously published method of histogram fitting [26]. Volumes are log-transformed to normalize population variance. Details have been described previously [26-30]. We dichotomized WMHI volume into high and low levels using the median as the cutoff.

We also categorized 1) peripheral vascular disease for those who had either CHD or HTN, 2) cerebrovascular diseases for those who had stroke, silent brain infarcts, CMB or WMHI > median, 3) vascular diseases for those had either peripheral or cerebrovascular diseases.

### Genome-wide association studies

To study the genotypes that were potentially impacted by circulating CD34+CD133+ progenitor cells for AD, GWAS were performed using the Framingham analytical pipeline. We conducted an interaction GWAS with log-transformed CD34+CD133+ cell frequency by using a logistic regression model for AD. The relationship between AD and interaction terms (CD34+CD133+ frequency and genome-wide SNP dosage) was tested using GEEPACK (logistic regression utilizing generalized estimating equations) while adjusting for age, sex, years of education, and the first 10 population principal components (PCs) for controlling ancestry/population substructures. Additive models were assumed. Only autosomal SNPs on chromosomes 1–22 were considered. A statically filtered set of dosage files for MACH imputation via the 1,000 genomes reference files from March 2012 of only samples of European ancestry was used. The filtering criterion was *r*^2^ ≥ 0.1. Moreover, only SNPs with minor allele frequency (MAF) ≥ 10% were chosen for the analysis. In addition, samples with a genotyping rate less than 97% and with an excess number of heterozygote observations or Mendelian errors were removed. Manhattan plot, Q-Q plot, and genomic control [31] were used for visualization and quality control, and LocusZoom [32] was used to present the regional information. Only *P* values < 5.0 × 10^−8^ were considered to be statistically significant for the SNP− CD34+CD133+ progenitor cell interactive effects for AD.

### Religious Orders Study/Memory and Aging Project

ROSMAP data were used to investigate the functions of the variants at the DNA methylation levels and gene expression levels and to explore their relationships with AD (https://dss.niagads.org/cohorts/religious-orders-study-memory-and-aging-project-rosmap/). ROSMAP participants have annual blood draws that provide a repository of stored serum, plasma, and cells. Clinical evaluation, self-report, and medication review are used to document medical conditions. Moreover, subjects are neurologically evaluated every year, and a review of all of the ante-mortem data at the time of death leads to a final clinical diagnosis for each participant; specifically, each individual receives a diagnosis of AD, mild cognitive impairment (MCI), or no cognitive impairment (NCI). The details of this methodology have been described on the website.

### Gene expression analysis and DNA methylation

We additionally explored the gene expression patterns across different AD brain regions by using Agora (https://agora.adknowledgeportal.org/genes), which included 9 brain regions, e.g., the anterior cingulate cortex (ACC), cerebellum (CBE), dorsolateral prefrontal cortex (DLPFC), frontal pole (FP), inferior frontal gyrus (IFG), posterior cingulate cortex (PCC), parahippocampal gyrus (PHG), superior temporal gyrus (STG), and temporal cortex (TCX). Two AD-related traits, e.g., BRAAK (neurofibrillary tangles) and CERAD (neuritic plaques), were also measured. In addition, GTEx Portal (https://gtexportal.org/home/) was used to study the associations between the selected SNPs and gene expression (eQTLs). The details of these procedures have been previously described [33].

We used Brain xQTLServe from ROSMAP (https://mostafavilab.stat.ubc.ca/xQTLServe/) to explore the associations between SNPs and DNA methylation (mQTLs). Genotype data were generated from 2,093 individuals of European descent. Of these individuals, DNA methylation (450K Illumina array, *n* = 468) was derived from postmortem frozen samples of a single cortical region, DLPFC. Gene expression data were generated by using RNA-sequencing (Illumina HiSeq) from the DLPFC of 494 individuals at an average sequence depth of 90 M reads. The details of this methodology have been previously described [34].

### Statistical analysis

Data were analyzed by using R version 4.2.1. The Cox models were applied to circulating cells including CD34+CD133+, CD34+, CD34+CD133−, CD34−CD133+, CD34−CD133−, or CD34+KDR+(VEGFR2), CD31+, CD31−, CD31+DIM, and CD31+lymphoid cells to examine the relationship between CD34+CD133+ or quartiles and dementia/AD diagnosis risk with adjustments for age, sex, years of education, *APOE* ε4, and different vascular diseases (Table S1). Only CD34+CD133+ progenitor cells were found to be associated with AD dementia.

Circulating CD34+CD133+ progenitor cells were first divided into four quartiles based on cell numbers. Quartiles of cell numbers were compared on the number of subjects, age at baseline, sex, years of education, *APOE* ε4, incident AD/dementia status, CHD, HTN, and different cerebrovascular diseases using analysis of variance (ANOVA), Chi-squared tests, and Kaplan-Meier survival analysis. We further stratified the subjects into the absence and the presence of different vascular pathologies and examined the relationship between CD34+CD133+ quartiles and dementia/AD diagnosis risk by using the Chi-squared test in each subgroup. Using ANOVA and Cuzick trend test, we also studied the relationship between CD34+CD133+ quartiles and brain volumes in the absence and presence of vascular diseases.

Furthermore, the Cox model was applied to the stratification of 1) no vascular diseases, 2) peripheral vascular diseases, and 3) cerebrovascular diseases with adjustments for age, sex, years of education, and *APOE* ε4. Additionally, the main effects and interaction effects (with CD34+CD133+) of GWAS-selected SNPs were obtained by using logistic regression and Cox proportional hazard regression adjusting for age, sex, years of education, *APOE* ε4, and PCs. These interaction effects were further assessed by applying stratification analyses of genotypes with Cox proportional hazard regression models and a Kaplan-Meier survival analysis.

## Results

### Determining which circulating endothelial subtypes associated with AD/dementia risk

We used the existing data from the FHS Gen2 cohort, which included 1,566 participants with an average age (mean ± SD) of 65.93 ± 8.91 years at exam 8 when circulating endothelial cells were measured at baseline, of which 46.36% were females. The concentrations (cell number per mL) and proportions of total PBMCs (%) of different EPCs and EMCs in this cohort were shown (Table 1, 2nd column).

There were 59 cases of incident AD after exam 8. Using Cox proportional hazards regression models, we examined the relationships between the proportions of different circulating cell populations (log-transformed) with following EPC marker combinations, CD34+CD133+, CD34+CD133−, CD34+, CD34−CD133+, CD34−CD133−, or CD34+KDR+ (VEGFR2), and AD or all-cause dementia risk. In addition, we studied EMCs including CD31+, CD31−, CD31+DIM, and CD31+lymphoid cells for AD risk using the same analyses. Among them, only CD34+CD133+ EPCs, but none of the other cell types, significantly impacted the risk of AD (HR = 0.64, *P* = 0.02) and all-cause dementia (HR = 0.63, *P* = 0.006) after adjusting for age, sex, years of education, *APOE* ε4, and vascular diseases (Table 1).

### Detailed characterizing the association between circulating CD34+CD133+ EPCs and the reduced risk for AD dementia

We further studied CD34+CD133+ EPCs in detail. The median concentration of CD34+CD133+ EPC counts was 2.61 × 10^−4^/mL; the median proportion of circulating CD34+CD133+ EPCs was 0.032% mononuclear cells measured at baseline. The relationship between CD34+CD133+ cell proportion (%) and AD risk or all-cause dementia risk was studied after adjusting for different covariates (Table 2). CD34+CD133+ EPC proportion was associated with reduced risk of AD or all-cause dementia without adding covariates (Model 1), and remained significant after adjusting for covariates of age, sex, years of education (Model 2), and the addition of *APOE* ε4 and vascular diseases (Model 3), showing that a higher frequency of circulating CD34+CD133+ EPCs decreased AD risk (HR = 0.64, 95% CI = 0.44–0.94, *P* = 0.02) and all-cause dementia risk (HR = 0.63, 95% CI = 0.45–0.87, *P* = 0.006). We also used the concentration (per mL) of CD34+CD133+ EPCs and found similar associations with reduced risk of AD dementia (Table S1).

**Figure 1.**
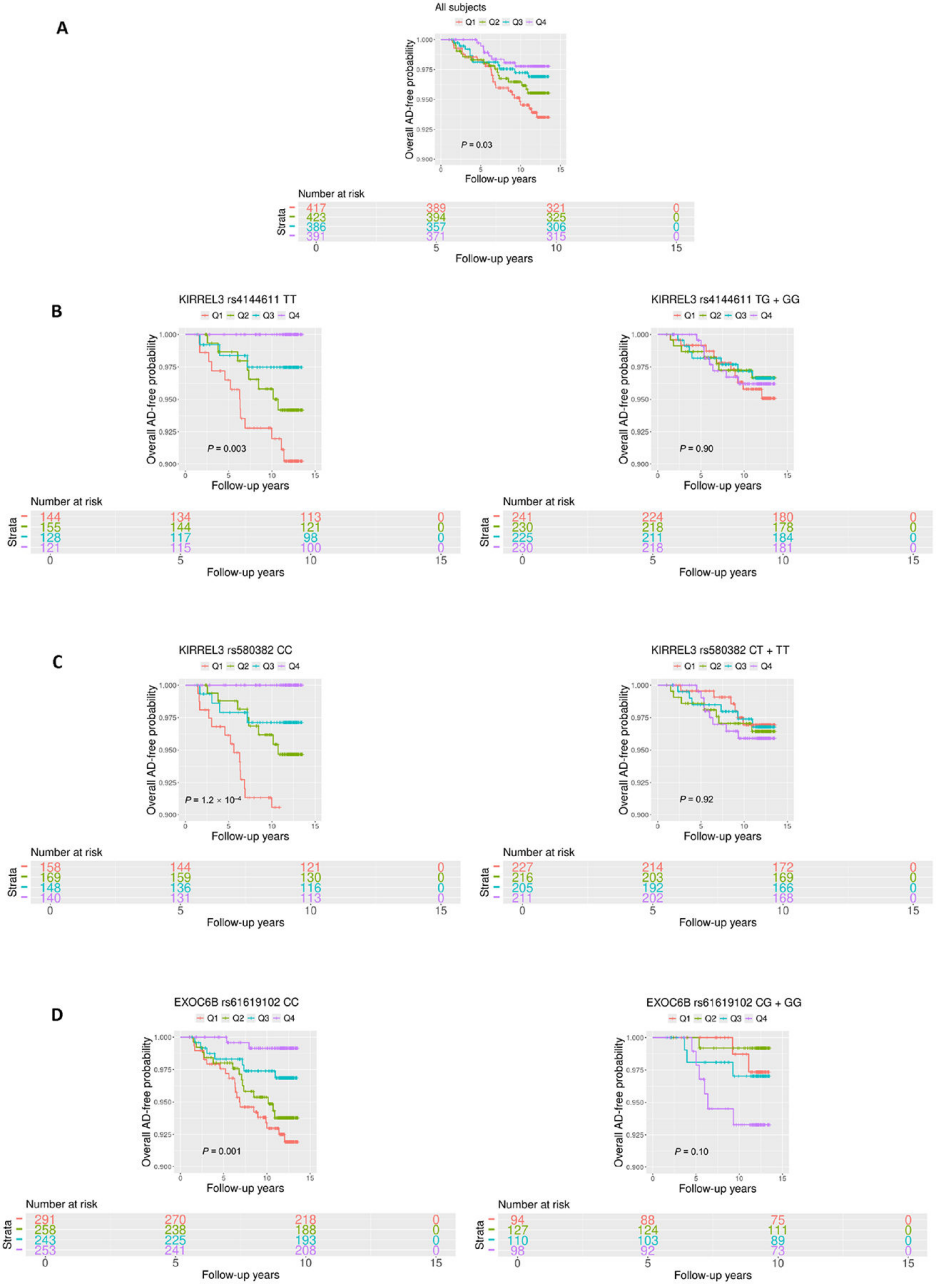
Survival analyses of circulating CD34+CD133+ cells for AD risk. Circulating the proportions (%) of CD34+CD133+ EPCs were divided into four quartiles based on the cell numbers (Q1 = 0.002–0.021; Q2 = 0.021–0.033; Q3 = 0.033–0.050; and Q4 = 0.050–0.609). Kaplan-Meier survival analysis was conducted to evaluate the survival-free time before AD over 14 years of follow-up to examine the relationship between CD34+CD133+ quartiles and AD incidence for: (A) all of the participants; (B) and (C) stratified based on the genotypes, (B) kirre like nephrin family adhesion molecule 3 (KIRREL3) rs4144611 major homozygotes TT *vs.* GG + TG; (C) KIRREL3 rs580382 major homozygotes CC *vs.* TT + CT, and (D) exocyst complex component 6B (EXOC6B) rs61619102 major homozygotes CC *vs.* GG + GC. *P* values are shown for statistical significance. Q: quartile

The participants were then divided into four quartiles based on the circulating CD34+CD133+ proportions (%) (Q1 = 0.002–0.020; Q2 = 0.020–0.032; Q3 = 0.032–0.049 and Q4 = 0.049–0.609). We found that younger (*P* = 0.007) and female (*P* = 0.002) participants were more likely to be in the higher CD34+CD133+ quartile (Table 3), with no significant differences in education and *APOE* ε4 carriers across the four quartiles. Vascular disease rates did not show statistical difference across the four CD34+CD133+ quartiles (Table 3). However, after adjusting for the confounders, CD34+CD133+ EPC cells were positively associated with HTN and body mass index (BMI) with statistical significance (Table S2). In contrast to positive associations with some vascular diseases, with an increase in the CD34+CD133+ EPC quartiles, the incident rates of AD (5.85% *vs.* 3.94% *vs.* 2.99% *vs.* 2.09%, *P* = 0.04) and all-cause dementia (7.66% *vs.* 5.56% *vs.* 4.41% *vs.* 2.86%, *P* = 0.02) decreased (Table 3). In parallel, Kaplan-Meier survival curves consistently revealed a reverse and a dose-dependent relationship between four increased CD34+CD133+ quartiles and decreased AD probability (*P* = 0.03, Figure 1).

### The relationship between CD34+CD133+ EPCs and reduced AD/dementia risk in the presence of peripheral and central vascular diseases

We hypothesized that CD34+CD133+ EPCs would show a significant association with AD dementia when vascular damage exists, in which the repair by the EPCs is needed. To approach this, the participants were stratified into those without any vascular disease *vs.* peripheral vascular diseases *vs.* cerebrovascular diseases. We found that increased CD34+CD133+ EPC frequency was significantly associated with decreased AD risk among those who had peripheral vascular diseases (HR = 0.62, 95% CI: 0.40–0.94, *P* = 0.02) or cerebrovascular disease (HR = 0.58, 95% CI: 0.35–0.96, *P* = 0.03, Table 2). Whereas this trend was not observed in individuals without any vascular diseases although the number of participants was small. Similar trends were observed for all-cause dementia risk among those individuals with peripheral vascular diseases (HR = 0.61, 95% CI: 0.43–0.88, *P* = 0.008) or with cerebrovascular disease (HR = 0.53, 95% CI: 0.35–0.81, *P* = 0.003). Again, similar to the proportion (%), the concentration of CD34+CD133+ EPCs per mL was found to have similar associations with AD dementia in the presence of peripheral or cerebrovascular diseases (Table S1).

**Figure 2.**
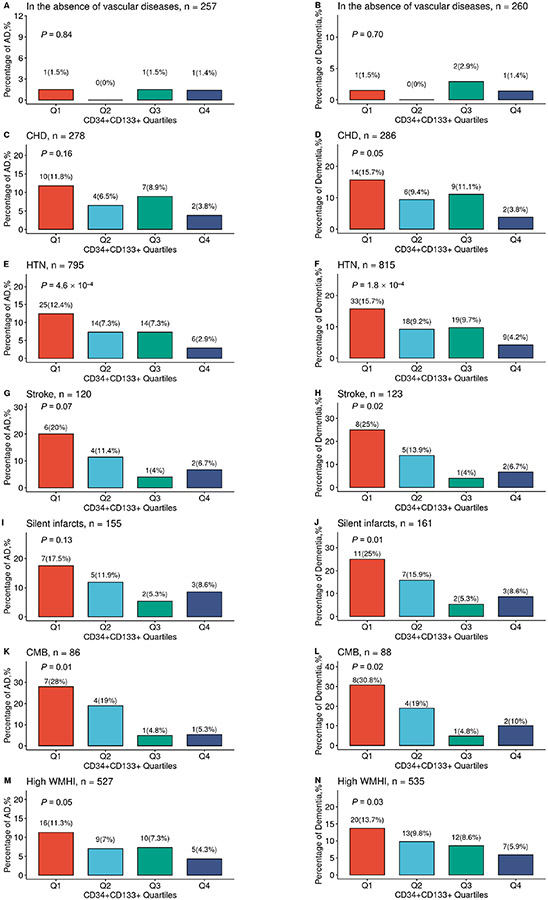
The relationship between circulating CD34+CD133+ cells and reduced AD risk in the presence of vascular diseases. The FHS participants were stratified into divided based on the CD34+CD133+ EPC proportion quartiles and further divided into those individuals with the absence or presence of different vascular diseases. In the absence of any peripheral and central vascular diseases (A, B), in the presence of CHD (C, D), HTN (E, F), stroke (G, H), silent infarcts (I, J), CMBs (K, L), and high WMHI (M, N), the numbers and percentages of incident AD (A, C, E, G, I, K, and M) and all-cause dementia (B, D, F, H, J, L, and N) across four CD34+CD133+ quartiles were examined by using Chi-squared test with indicated *P* values. Q: quartile

We then examined this relationship in each specific vascular disease (Figure 2A-N). We found that increased CD34+CD133+ quartiles had a significantly dose-dependent association with decreased AD risk among participants who had HTN (*P* = 4.6 × 10^−4^, Figure 2E) and CMB (*P* = 0.01, Figure 2K). Increasing CD34+CD133+ EPC quartiles also had a reverse relationship with the risk of all-cause dementia among those who had CHD (*P* = 0.05, Figure 2D), HTN (*P* = 1.8 × 10^−4^, Figure 2F], stroke (*P* = 0.02, Figure 2H), silent infarct (*P* = 0.01, Figure 2J), CMB (*P* = 0.02, Figure 2L), or had a high WMHI level (*P* = 0.03, Figure 2N].

In this FHS cohort, compared to those participants without any vascular diseases (0.78%), participants with peripheral vascular diseases, including CHD (8.27%, *P* = 4.1 × 10^−5^) and HTN (7.42%, *P* = 7.5 × 10^−5^), and participants with cerebrovascular diseases, including clinical diagnoses of stroke (10.83%, *P* = 3.3 × 10^−6^), silent infarct (10.97%, *P* = 1.8 × 10^−6^), CMB (15.12%, *P* = 1.8 × 10^−8^), and high volume of WMHI (7.59%, *P* = 7.0 × 10^−5^), had significantly higher rates of incident AD. A similar but stronger trend was observed for the incident rates of all-cause dementia in participants with these vascular diseases. However, we did not find the interactive effects between CD34+CD133+ EPCs or the other cell types and the combined vascular diseases for the risk of AD dementia (Table S3), probably due to that the proportion of those without any vascular diseases was small (*n* = 256) with only 3 cases of AD onset so that we did not have power to detect the interactive effects.

### Characterizing the relationship between CD34+CD133+ EPCs and brain volumes

To study the relationship between CD34+CD133+ EPCs and brain volumes, we used a subsample (*n* = 1,172) with available brain MRI data. Since white matter pathologies in the brain are linked with vascular diseases [35], we examined brain gray *versus* white volumes of different brain lobes across four CD34+CD133+ proportion quartiles in the absence and the presence of vascular diseases by using ANOVA and Cuzick trend test (Table 4). We examined 11 brain regions and found that an increase in the CD34+CD133+ EPC quartiles was associated with bigger cerebrum white matter, but not grey matter, volumes in the presence of vascular diseases (*P* = 0.004), especially related to the white matter volumes in frontal lobe (*P* = 0.007) and temporal lobe (*P* = 0.046). In addition, the higher CD34+CD133+ EPC quartiles, the bigger volume of hippocampus in the presence of vascular diseases (*P* = 0.023). We also studied these associations in the presence of particular vascular diseases. In the presence of HTN, CD34+CD133+ EPCs were significantly associated with the brain volumes of cerebrum white matter (*P* = 0.0006), frontal white matter (*P* = 0.004), temporal white matter (*P* = 0.014), and hippocampus (*P* = 0.0016, Table 4). This finding was consistent with the relationship between CD34+CD133+ EPCs and reduced AD risk in HTN (Figure 2E). In the presence of CMB, CD34+CD133+ EPCs were significantly associated with frontal gray matter and hippocampus, but the relationships were not linear.

### The effect of CD34+CD133+ EPCs on AD risk in the KIRREL3 and EXOC6B homozygous genotype carriers

**Figure 3.**
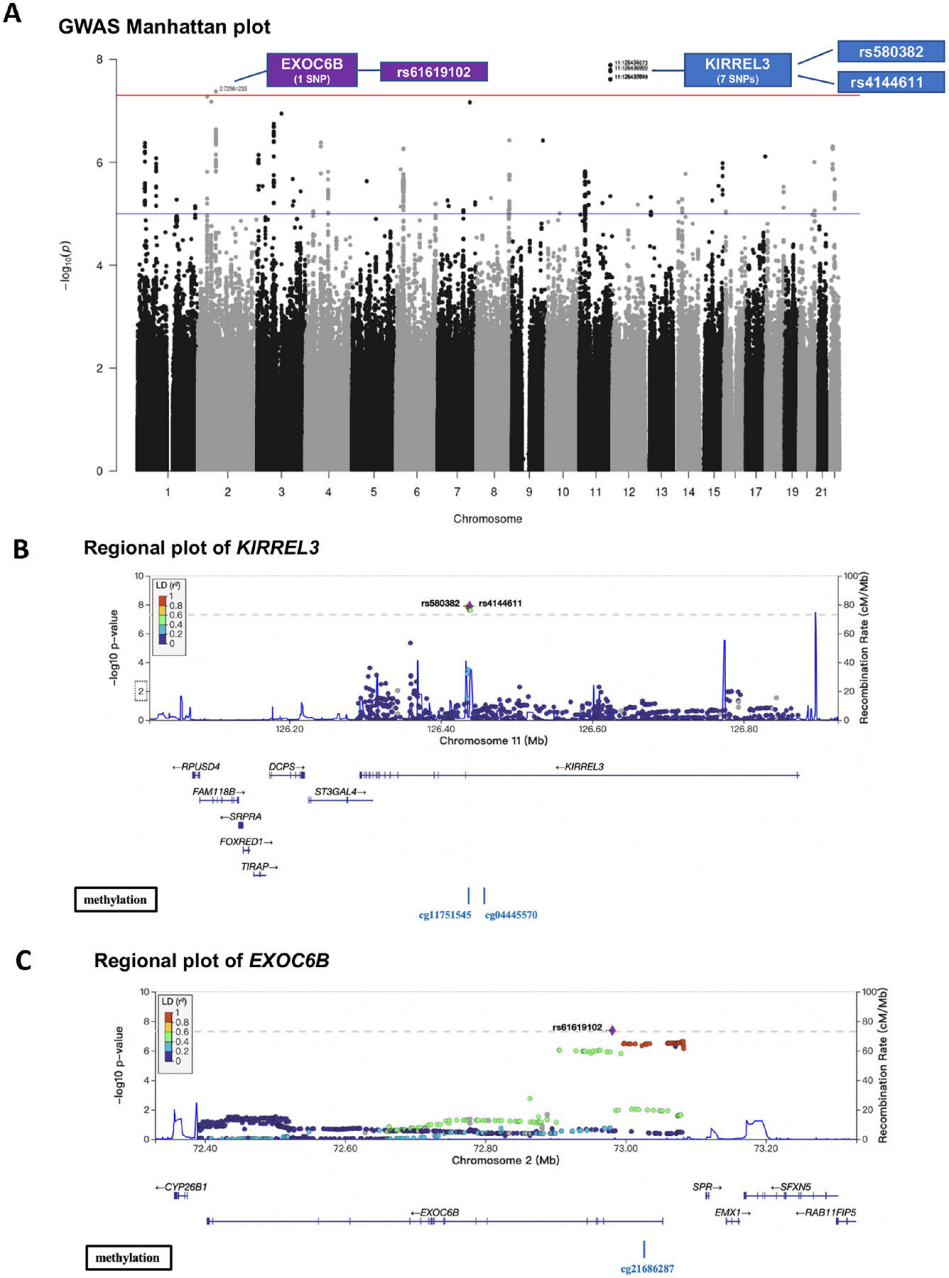
GWAS on the interactive effects of genetic factors and circulating CD34+CD133+ cells on AD risk. The FSH analytical pipeline was used to perform GWAS. The interaction between genome-wide SNP dosage and log-transformed proportions (%) of circulating CD34+CD133+ EPCs for AD risk was tested by using the GEEPACK model (logistic regression utilizing generalized estimating equations) adjusting for age, sex, years of education, and the first 10 PCs. As shown in the Manhattan plot, 8 SNPs showed significant interactions with CD34+CD133+ frequency on AD risk (A). Seven of the identified SNPs with a *P* value < 5.0 × 10^−8^ originated from gene *KIRREL3* on chromosome 11 (rs580382 and rs4144611 were selected for further analysis), and 1 of them originated from *EXOC6B* (chromosome 2, rs61619102). Only *r*^2^ ≥ 0.1 and SNPs with MAF ≥ 10% were considered. Manhattan plot (A) was used for visualization to identify the genotypes of two genes (*KIRREL3* and *EXOC6B*) that had interactive effects with CD34+CD133+ EPCs for AD risk. Gene structures, surrounding regions, and methylation sites of *KIRREL3* (B) and *EXOC6B* (C) are presented by using LocusZoom

Studies have shown that brain endothelial damage increases AD pathogenesis in the brain in certain genotype carriers [6], therefore, we hypothesized that peripheral circulating CD34+CD133+ EPCs may reduce AD risk in particular genotypes. To test this hypothesis, we first conducted a GWAS for AD with the interaction term between CD34+CD133+ cell frequency and SNP as predictor using a logistic regression model. For SNPs passing a genome-wide significance (*P* < 5.0 × 10^−8^), a Cox regression model was also applied to validate the interaction effects on AD incidence. As depicted in the Manhattan plot, 8 SNPs showed significant interactions with CD34+CD133+ frequency for AD risk (Figure 3A). Seven of the 8 SNPs with a *P* value < 5.0 × 10^−8^ were located in gene *KIRREL3* on chromosome 11 (two of the 7 SNPs selected for further analysis: rs580382 and rs4144611) and 1 of the 8 SNPs was in gene *EXOC6B* (chromosome 2, rs61619102) (Figure 3A, Figure S2). These associations are supported by evidence with surrounding SNPs (Figures 3B and 3C, Table S4). Notably, for these 3 SNPs, although their main SNP effects were not associated with AD risk (*P* > 0.40), their interactive effects with CD34+CD133 cells were significantly associated with AD after adjusting for age, sex, years of education, *APOE* ε4, and PCs using both logistic and Cox regression analyses (Table 5).

Consistently, in genotype stratification analysis adjusting for age, sex, years of education, *APOE* ε4, and PCs, we found that CD34+CD133+ frequency was negatively associated with AD incidences among the homozygotes of *KIRREL3* rs4144611 TT carriers (HR = 0.29, 95% CI: 0.15–0.57, *P* < 0.001), *KIRREL3* rs580382 CC carriers (HR = 0.31, 95% CI: 0.17–0.57, *P* < 0.001), and *EXOC6B* rs61619102 CC carriers (HR = 0.49, 95% CI: 0.31–0.75, *P* < 0.001) (Table S5). Further examination of these findings revealed a dose-dependent effect of increased CD34+CD133+ cell quartiles on the reduction in AD risk among *KIRREL3* rs4144611 TT or rs580382 CC homozygotes as well as in *EXOC6B* rs61619102 CC homozygotes (Table 6, Table S6, and Table S7). In contrast, these impacts from CD34+CD133+ cells on AD risk were not present in the counterpart genotypes of these 3 SNPs, e.g., *KIRREL3* rs4144611 either TG or GG; rs580382 either CT or TT; as well as *EXOC6B* rs61619102 either GC or GG carriers (Tables S5, S6, and S7).

These relationships were also observed in Kaplan-Meier survival curves. Among the homozygotes of *KIRREL3* rs4144611 TT carriers (*P* = 0.003, Figure 1B), *KIRREL3* rs580382 CC carriers (*P* = 1.2 × 10^−4^, Figure 1C), and *EXOC6B* rs61619102 CC carriers (*P* = 0.001, Figure 1D), higher CD34+CD133+ quartiles corresponded to a lower incidence of AD development and difference in AD-free probability with statistical significance. Again, among their counterpart genotype carriers (*KIRREL3* rs4144611 GG + TG or rs580382 TT + CT carriers and *EXOC6B* rs61619102 GG + GC carriers), there were no such relationships between CD34+CD133+ quartiles and AD or all-cause dementia risk. Additionally, unlike for AD dementia risk, we did not find associations of these genotypes or the interactive effects of these genotypes and CD34+CD133+ cells for any vascular disease (data not shown).

### Exploration of *KIRREL3* and *EXOC6B* genes in AD

To address why these two genes could interact with CD34+CD133+ EPCs for AD risk, we explored if the mRNA expressions level of gene *KIRREL3* and *EXOC6B* are associated with AD pathology. We first used the RNA-seq dataset generated from the DLPFC region and monocytes in the ROSMAP study. As shown in Figure S3, brain *KIRREL3* expression was associated with AD (*P* = 0.02), but peripheral *KIRREL3* expression was not (Figure S3A). Compared to controls, brain expression level of *KIRREL3* was lower in AD brains specifically in the TCX (*P* = 2.7 × 10^−5^) regions (Figure S3B). To investigate the relationship between *KIRREL3* SNPs and lower mRNA levels in the AD brain, we investigated the epigenetics of *KIRREL3* SNPs and found that the methylation site cg11751545 was consistently negatively correlated with *KIRREL3* gene expression in DLPFC (Figure S4A). Two *KIRREL3* genotypes (rs4144611 GG + GT or rs580382 TT + CT) were negatively associated with DNA methylation level of two CpG sites within *KIRREL3* (cg11751545; cg04445570; *P* < 0.05) (Figure 3B, Figure S4B). Based on these results, we hypothesized that the rescue effect of CD34+CD133+ EPCs may reduce AD risk only in vulnerable *KIRREL3* rs4144611 TT carriers (Table 5 and Figure 1B and Figure S3C) and rs580382 CC carriers (Tables 5 and 6) because the low brain expression of *KIRREL3* is regulated by the high brain methylation of cg11751545 in these carriers.

While peripheral monocyte expression of *EXOC6B* was significantly and positively associated with AD (*P* < 0.001), the brain expression of *EXOC6B* was not associated with AD after Bonferroni correction (Figure S5A). Consistently, *EXOC6B* rs61619102 G allele was positively associated with *EXOC6B* gene expression as an eQTL compared to C allele in the following two types of peripheral tissues: visceral and subcutaneous adipose tissues (Figure S5B). Thus, we reasoned that the impact of *EXOC6B* polymorphisms on AD was mainly elicited through the peripheral system and that the *EXOC6B* rs61619102 homozygous genotype (CC) itself (which was associated with a low level of expression in the peripheral system) interacted with circulating CD34+CD133+ EPCs to reduce AD risk (Table 5; Figure 1D and Figure S5C).

## Discussion

As peripheral and central vascular diseases increase AD risk, there may be some protective factors *in vivo* antagonizing or rescuing cerebrovascular pathologies to reduce the risk of AD. Our longitudinal cohort found that circulating CD34+CD133+ EPCs were negatively associated with AD risk, suggesting a protective role against AD risk via repairing damaged endothelia in cerebrovasculature. Consistently with our finding, circulating EPCs with the co-expression of CD34, CD133, and KDR have pronounced tube formation activity *in vitro*, and strong reendothelialization or neovascularization capacity *in vivo* [36].

Among different endothelial cells we examined, only circulating CD34+CD133+ EPCs showed dose-dependent protective effects against AD development, especially in the presence of HTN and CMB (Tables 1 and 2; Figure 2). As brain endothelia marked with CD31 is associated with AD pathology [6], it is likely that there is a balance between the cellular damage/pathology of mature endothelia and a repair by circulating EPCs for in the AD pathological process. Supporting our findings, one clinical study (*n* = 40) found that AD patients had a lower level of circulating CD34+CD133+ EPCs than controls [13]. Human cord blood CD34+CD133+ cells have been shown to reduce the apoptosis of endothelial and ganglion cells and increase the number of surviving CD31+ EMCs in the retina after radiation damage [37].

CD34 (also known as sialomucin) is a transmembrane protein expressed on early hematopoietic and vascular-associated tissues [38]. CD133 is a stem cell marker, and peripheral blood-derived CD133+ cells can further differentiate into hematopoietic, endothelial, and muscle cells *in vivo* and *in vitro* [39]. Another cross-sectional study reported that circulating CD34+ cells were higher, not lower, in AD than in controls [40]. In our study, without the other expression, the relationship between EPCs with either CD34+ or CD133+ expression alone and AD risk showed a trend but did not reach statistical significance (Table 1). It is shown that the leukocyte antigen CD34 is localized to the vascular endothelium throughout the human brain [41], suggesting that CD34+ cells are mixed with endothelial progenitors and leukocytes, but CD34+CD133+ cells are specific to be endothelial progenitors. On the other hand, CD133+ donor cells were detected in several vessels near areas of regeneration, where they expressed human VE-cadherin and CD31, which are biomarkers of mature endothelia [42]. Based on our data and others, it is likely that CD34+CD133+ EPCs are the specific subtype of progenitors that repair damaged brain endothelia and rescue AD risk.

CD34+CD133+ EPCs were associated with reduced AD risk (Table 2 and Figure 2) and positively associated with brain white matter volumes (Table 4), particularly in the presence of HTN and CMB. Cerebrovascular diseases are complex diseases involving different or multiple cell type pathologies and endothelial pathology is one of them [43, 44]. One study shows that autologous circulating endothelial precursor cells may have the potential for the treatment of ischemic diseases [45]. Some studies have shown that the relationship between CD34+CD133+ EPCs and cerebral small vessel disease (CSVD) is positive [46, 47], but another one shows a negative relationship [48]. It is reported that the role of cerebral microvasculature pathology in AD is likely through dysfunctional endothelia (reviewed by Dalkara and Alarcon-Martinez) [49], suggesting that endothelial damage/dysfunction in cerebral pathology is an important element for the relationship between cerebrovascular risk and AD pathogenesis.

Our study (Table 3) and others have shown that increased age may alter the availability of or decrease the number of circulating CD34+CD133+ EPCs [50-52]. We found that HTN and BMI were positively associated with CD34+CD133+ EPCs in FHS (Table S2), but other studies show that aging diseases like cardiovascular diseases [53] and diabetes [54] and the absence of healthy aging are associated with decreased CD34+CD133+ EPCs. Importantly, it is noted that an increased number of circulating CD34+CD133+ EPCs is associated with better outcomes of physical function [55] and cardiovascular diseases [53]. Although we found that females have more CD34+CD133+ EPCs in the FHS (Table 3), another study found that females have a lower number of CD34+CD133+ EPCs than males [56]. The discrepancies could be attributed to different age and disease periods in different cohorts.

GWAS analyses revealed that CD34+CD133+ cells differentially affected AD protection in the genotypes of two genes (*KIRREL3* and *EXOC6B*) (Figure 3; Tables 5 and 6). *KIRREL3* as an adhesion molecule is found to play roles in morphology change and migration of muscle progenitor cells [57, 58], and it is also involved in the differentiation of hematopoietic stem cells [59, 60], *KIRREL3* has been shown to mediate neuron synapse formation and cell adhesion/guidance [61-63], as well as cell differentiation [57, 58], *EXOC6B* has the exocyst functions in the niche to promote germline stem cell (GSC) progeny differentiation in the *Drosophila* ovary by directly regulating EGFR membrane trafficking and signaling [64]. In the literature, there is no report on these genes for CD34+CD133+ EPCs yet. However, since both genes are shown to be involved in differentiations of stem and progenitor cells, it is possible that the *KIRREL3* rs4144611 TT or rs580382 CC, and *EXOC6B* rs61619102 CC genotypes (Figure 1B-D) rescue endothelial pathology in AD via promoting circulating CD34+CD133+ EPCs to differentiate into brain endothelia.

Both *KIRREL3* and *EXOC6* (the high homolog of *EXOC6B*) have been shown to be risk genes for AD. One meta-analysis of GWAS with a large number of participants showed that SNPs of *KIRREL3* are a genetic risk factor for AD [65, 66]. The loss of *KIRREL3* leads to changed axon organization, as well as male-male aggression and cognitive impairment, in mice [67-69]. In noncarriers of *APOE* ε4, the *EXOC6* gene was found to be associated with AD risk [70]. Interestingly, both *KIRREL3* [58, 62, 71-77] and *EXOC6B* [78-80] genes are also related to autism spectrum disorder (ASD), another cognitive disease with a young age of onset. Like neurodegenerative diseases, ASD is also associated with cerebral hypoperfusion [81], suggesting the presence of common brain endothelial pathologies in both diseases.

This study had some limitations. The FHS study was based on a non-Hispanic White population and more large cohorts with different ethnicities are needed to replicate the finding that CD34+CD133+ EPCs are associated with reduced risk of AD dementia. Moreover, although we had a one-time point of CD34+CD133+ cell measurement, longitudinal measurements are necessary to characterize the change of CD34+CD133+ EPCs during aging and the disease process. Future clinical trials with circulating CD34+CD133+ EPCs are key to proving that it can be therapeutic for AD prevention. Nevertheless, our study sheds some light for increasing circulating CD34+CD133+ endothelial progenitors to reduce AD risk, especially among those individuals with vascular diseases and the vulnerable *KIRREL3* and *EXOC6B* genotypes.

## Availability of data and materials

The FHS data is available at dbGaP (https://www.ncbi.nlm.nih.gov/projects/gap/cgi-bin/study.cgi?study_id=phs000007.v33.p14) upon reasonable request/application. The ROSMAP data is available at the AD Knowledge Portal (https://adknowledgeportal.org). The other datasets that support the findings of this study are available from the corresponding author upon reasonable request.

## Supplementary Material

supplement materials

## Figures and Tables

**Table 1.** Examination of the relationship between different circulating progenitor or mature endothelial cells and the risk of AD/dementia by using Cox proportional hazards regression models after adjusting for covariates

Circulating cells	Number/mL (%) Median [Q1, Q3]	AD HR (95% CI)	*P* value	All-cause dementia HR (95% CI)	*P* value
CD34+CD133+	2.61 × 10^4^ [1.65 × 10^4^, 4.39 × 10^4^]	0.64 (0.44, 0.94)	0.02*	0.63 (0.45, 0.87)	0.006*
	0.031 [0.020, 0.049]	*n* = 1,340		*n* = 1,362	
CD34+CD133−	2.13 × 10^4^ [1.38 × 10^4^, 3.19 × 10^4^]	0.79 (0.49, 1.27)	0.33	0.74 (0.50, 1.10)	0.14
	0.025 [0.017, 0.038]	*n* = 1,340		*n* = 1,362	
CD34−CD133+	3.06 × 10^4^ [2.33 × 10^4^, 4.15 × 10^4^]	0.56 (0.28, 1.14)	0.11	0.81 (0.46, 1.43)	0.47
	0.037 [0.028, 0.047]	*n* = 1,340		*n* = 1,362	
CD34−CD133−	8.59 × 10^7^ [6.91 × 10^7^, 10.56 × 10^7^]	342.92 (0.62, 1.91 × 10^5^)	0.07	62.05 (0.37, 1.03 × 10^4^)	0.11
	99.90 [99.90, 99.90]	*n* = 1,340		*n* = 1,362	
CD34+	6.36 × 10^4^ [4.44 × 10^4^, 9.51 × 10^4^]	0.62 (0.36, 1.04)	0.07	0.61 (0.39, 0.96)	0.03*
	0.076 [0.053, 0.110]	*n* = 1,415		*n* = 1,436	
CD34+/KDR+	3.38 × 10^6^ [1.96 × 10^6^, 5.71 × 10^6^]	0.91 (0.65, 1.28)	0.59	0.91 (0.68, 1.23)	0.55
	3.96 [2.33, 6.58]	*n* = 1,416		*n* = 1,437	
CD31+/CD45−	5.14 × 10^5^ [2.93 × 10^5^, 8.78 × 10^5^]	0.85 (0.62, 1.17)	0.32	0.85 (0.65, 1.11)	0.24
	0.60 [0.36, 1.01]	*n* = 1,431		*n* = 1,453	
CD31+	1.73 × 10^7^ [1.19 × 10^7^, 2.42 × 10^7^]	0.86 (0.43, 1.70)	0.65	0.74 (0.42, 1.31)	0.30
	20.24 [15.12, 26.00]	*n* = 1,400		*n* = 1,421	
CD31−	3.11 × 10^7^ [2.35 × 10^7^, 4.02 × 10^7^]	1.46 (0.69, 3.08)	0.32	1.64 (0.85, 3.16)	0.14
	39.39 [29.98, 47.93]	*n* = 1,400		*n* = 1,421	
CD31+DIM	3.36 × 10^7^ [2.38 × 10^7^, 4.43 × 10^7^]	0.73 (0.32, 1.66)	0.45	0.73 (0.36, 1.48)	0.38
	39.03 [31.99, 46.82]	*n* = 1,400		*n* = 1,421	
CD31+lymphoid	6.57 × 10^7^ [5.38 × 10^7^, 8.22 × 10^7^]	1.91 (0.16, 22.65)	0.61	2.76 (0.32, 24.12)	0.36
	79.76 [74.00, 84.88]	*n* = 1,400		*n* = 1,421	

The concentration (cell number/mL) and the proportion (%) of different circulating endothelial cell (EPCs) and EMCs are illustrated in the 2nd column. Cox proportional hazards regression models were used to study the relationship between the proportions (%) of different subtypes of EPCs and EMCs (log-transformed) and the risk of AD or all-cause dementia after adjusting for age, sex, years of education, *APOE* ε4, and vascular diseases. HR with 95% CI with *P* values is shown. * *P* value significant < 0.05; CI: confidence interval; HR: hazard ratio; Q: quartile

**Table 2.** Detailed examination of the relationship between CD34+CD133+ (log-transformed) and the risk of AD/dementia risk

CD34+CD133+ cells plus covariates	AD		All-cause dementia	
	HR (95% CI)	*P* value	HR (95% CI)	*P* value
Model 1: no covariates	0.57 (0.40, 0.81)	0.002*	0.57 (0.42, 0.77)	0.003*
	*n* = 1,617		*n* = 1,640	
Model 2: age, sex, years of education	0.64 (0.45, 0.93)	0.02*	0.64 (0.47, 0.87)	0.005*
	*n* = 1,617		*n* = 1,640	
Model 3: Model 2 + APOE ε4 + vascular diseases	0.64 (0.44, 0.94)	0.02*	0.63 (0.45, 0.87)	0.006*
	*n* = 1,340		*n* = 1,362	
Model 3 after stratification				
No vascular diseases^a^	1.23 (0.15, 10.18)	0.85	1.12 (0.19, 6.56)	0.90
	*n* = 256		*n* = 257	
Peripheral vascular diseases^b^ only	0.62 (0.40, 0.94)	0.02*	0.61 (0.43, 0.88)	0.008*
	*n* = 832		*n* = 850	
Cerebrovascular diseases^c^ only	0.58 (0.35, 0.96)	0.03*	0.53 (0.35, 0.81)	0.003*
	*n* = 628		*n* = 641	

Cox proportional hazards regression models were used to study the relationship between log-transformed CD34+CD133+ cell frequency (%) and the risk of AD or all-cause dementia after adjusting for the covariates. HR with 95% CI with *P* values is shown. Model 1: simple association without confounders; Model 2: adjusting for age, sex, and education; Model 3: Model 2 + *APOE* ε4 + vascular diseases. ^a^ Model 3 after the stratification as no vascular diseases for those with no CHD, no HTN, no stroke, no silent infarct, no CMB, and low level of WMHI; ^b^ Model 3 after the stratification as peripheral vascular diseases for those with CHD or HTN; ^c^ Model 3 after the stratification as cerebrovascular diseases for those with stroke, silent infarct, CMB, or high level of WMHI; * *P* value significant < 0.05

**Table 3.** Characteristics of the study sample based on circulating CD34+CD133+ EPC quartiles

Characteristics	Overall	Blood CD34+CD133+ EPCs frequency, %				
	[0.002, 0.609] (*n* = 1,566)	1st Q [0.002, 0.020] (*n* = 410)	2nd Q [0.020, 0.032] (*n* = 406)	3rd Q [0.032, 0.049] (*n* = 368)	4th Q [0.049, 0.609] (*n* = 382)	*P* value
Age, (mean ± SD), y	65.93 ± 8.91	66.93 ± 9.08	66.15 ± 9.03	65.74 ± 8.60	64.78 ± 8.79	0.007^a^*
Sex, Female, *n* (%)	726 (46.36%)	168 (40.98%)	173 (42.61%)	184 (50.00%)	201 (52.62%)	0.002^b^*
Education, (mean ± SD), y	14.10 ± 2.50	14.07 ± 2.55	14.12 ± 2.61	14.16 ± 2.49	14.07 ± 2.32	0.94^a^
*APOE* ε4 carrier^c^, *n* (%)	342 (21.84%)	85 (20.73%)	87 (21.43%)	94 (25.54%)	76 (19.90%)	0.25^b^
Peripheral vascular diseases						
CHD, *n* (%)	260 (16.60%)	80 (19.51%)	64 (15.76%)	67 (18.21%)	49 (12.83%)	0.06^b^
HTN, *n* (%)	755 (48.21%)	194 (47.32%)	200 (49.26%)	160 (43.48%)	201 (52.62%)	0.09^b^
Cerebrovascular diseases						
Stroke, *n* (%)	107 (6.83%)	27 (6.59%)	31 (7.64%)	22 (5.98%)	27 (7.07%)	0.82^b^
Silent brain infarcts, *n* (%)	146 (9.32%)	36 (8.78%)	42 (10.34%)	34 (9.23%)	34 (8.90%)	0.90^b^
CMB, *n* (%)	79 (7.68%)	21 (8.05%)	20 (7.41%)	19 (7.98%)	19 (7.34%)	0.99^b^
WMHI, cm^3^, (mean ± SD)	4.45 ± 7.54	5.16 ± 9.03	4.58 ± 8.32	4.09 ± 5.45	3.92 ±6.61	0.25^a^
Incidence						
AD, *n* (%)	59 (3.77%)	24 (5.85%)	16 (3.94%)	11 (2.99%)	8 (2.09%)	0.04^b^*
Dementia, *n* (%)	82 (5.16%)	32 (7.66%)	21 (5.56%)	18 (4.41%)	11 (2.86%)	0.02^b^*

The participants were divided based on CD34+CD133+ cell frequency (%) quartiles and compared. ^a^ ANOVA test *P* value; ^b^ Chi-squared test *P* value;^c^ *APOE* ε4 = ε34 + ε44; * *P* value significant < 0.05; Q: quartile; y: years

**Table 4.** Comparisons of brain volumes across circulating CD34+CD133+ EPC quartiles

Brain volumes^a^	Stratification	Blood CD34+CD133+ EPCs frequency, %					
Mean (SD)		1st Q	2nd Q	3rd Q	4th Q	ANOVA	Cuzick trend test
		[0.002, 0.021]	[0.021, 0.033]	[0.033, 0.050]	[0.050, 0.609]	*P* value^b^	*P* value^b^
		(*n* = 302)	(*n* = 310)	(*n* = 271)	(*n* = 289)		
Cerebrum gray matter	No vascular diseases	40.181 (1.793)	40.276 (1.340)	40.273 (1.554)	40.285 (1.441)	0.978	0.728
	Vascular diseases	38.891 (1.762)	39.056 (1.844)	39.282 (1.898)	38.890 (1.840)	0.088	0.468
Cerebrum white matter	No vascular diseases	36.789 (2.255)	37.264 (1.620)	37.303 (2.027)	37.273 (2.151)	0.405	0.296
	Vascular diseases	36.088 (2.669)	35.975 (2.354)	35.980 (2.595)	36.814 (2.709)	0.001*	0.004*
Frontal gray matter	No vascular diseases	14.160 (0.942)	14.357 (0.678)	14.305 (0.690)	14.326 (0.714)	0.457	0.392
	Vascular diseases	13.714 (0.788)	13.792 (0.781)	13.907 (0.766)	13.739 (0.833)	0.056	0.360
Frontal white matter	No vascular diseases	13.479 (1.058)	13.670 (0.711)	13.656 (0.849)	13.694 (0.968)	0.500	0.209
	Vascular diseases	13.274 (1.112)	13.232 (1.077)	13.273 (1.164)	13.585 (1.232)	0.003*	0.007*
Parietal gray matter	No vascular diseases	7.940 (0.511)	7.985 (0.430)	7.983 (0.473)	7.984 (0.453)	0.931	0.744
	Vascular diseases	7.673 (0.477)	7.734 (0.514)	7.725 (0.495)	7.616 (0.543)	0.054	0.215
Parietal white matter	No vascular diseases	7.297 (0.606)	7.350 (0.485)	7.372 (0.549)	7.423 (0.514)	0.591	0.298
	Vascular diseases	7.211 (0.650)	7.169 (0.586)	7.109 (0.590)	7.297 (0.689)	0.017*	0.252
Occipital gray matter	No vascular diseases	4.856 (0.421)	4.795 (0.377)	4.807 (0.395)	4.851 (0.395)	0.755	0.905
	Vascular diseases	4.628 (0.468)	4.643 (0.435)	4.692 (0.482)	4.655 (0.458)	0.501	0.437
Occipital white matter	No vascular diseases	4.284 (0.384)	4.334 (0.373)	4.300 (0.418)	4.273 (0.400)	0.828	0.683
	Vascular diseases	4.194 (0.453)	4.148 (0.417)	4.196 (0.421)	4.250 (0.445)	0.099	0.161
Temporal gray matter	No vascular diseases	9.950 (0.437)	9.871 (0.519)	9.874 (0.494)	9.842 (0.471)	0.600	0.215
	Vascular diseases	9.566 (0.573)	9.615 (0.609)	9.629 (0.620)	9.618 (0.571)	0.683	0.296
Temporal white matter	No vascular diseases	6.251 (0.501)	6.249 (0.387)	6.345 (0.425)	6.252 (0.463)	0.543	0.907
	Vascular diseases	5.943 (0.591)	5.931 (0.513)	5.942 (0.572)	6.065 (0.576)	0.038*	0.046*
Hippocampus	No vascular diseases	0.550 (0.045)	0.562 (0.045)	0.557 (0.045)	0.550 (0.050)	0.393	0.813
	Vascular diseases	0.529 (0.050)	0.537 (0.052)	0.536 (0.049)	0.541 (0.047)	0.091	0.023*
HTN							
Cerebrum white matter		35.842 (2.835)	35.805 (2.381)	36.011 (2.617)	36.815 (2.677)	0.002*	0.0006*
Frontal white matter		13.177 (1.179)	13.150 (1.033)	13.257 (1.140)	13.546 (1.204)	0.009*	0.004*
Temporal white matter		5.906 (0.612)	5.915 (0.547)	5.942 (0.590)	6.075 (0.581)	0.039*	0.014*
Hippocampus		0.523 (0.047)	0.534 (0.053)	0.532 (0.053)	0.542 (0.046)	0.015*	0.0016*
CMBs							
Frontal gray matter		13.307 (0.684)	13.428 (0.907)	13.987 (0.700)	13.572 (0.692)	0.022*	0.044*
Hippocampus		0.510 (0.040)	0.538 (0.060)	0.545 (0.038)	0.537 (0.048)	0.061	0.040*

The participants who had brain volume measurements were divided based on CD34+CD133+ cell frequency (%) quartiles and further divided into those who did not *vs.* had vascular diseases. ANOVA and Curick trend test were used to compare MRI brain volumes, gray matter and white matter volumes, across four quartiles in the absence and the presence of vascular diseases. ^a^ Regional brain volumes were divided by cerebrum cranial volume; ^b^ Bonferroni significance = 0.005 (0.05/11); * *P* value significant < 0.05

**Table 5.** Interactions between SNPs of *KIRREL3* or *EXOC6B* and CD34+CD133+ on AD risk in FHS

Gene locus	Chr:Pos (GRCh37)	Major allele	Minor allele	dbSNP ID	Function	MAF	AD risk					
							Main effect of SNP^a^		Interaction effect (GEEPACK) (SNP with CD34+CD133+)^b^		Interaction effect (Cox) (SNP with CD34+CD133+)^c^	
							OR (95% CI)	*P* value	OR (95% CI)	*P* value	HR (95% CI)	*P* value
*KIRREL3*	chr11:126434673	T	G	rs4144611	Intron	0.39	1.12 (−0.15, 0.37)	0.40	5.52 (3.18, 9.56)	1.3 × 10^−8^*	2.71 (1.67, 4.40)	5.61 × 10^−7^*
*KIRREL3*	chr11:126438111	C	T	rs580382	Intron	0.35	1.07 (−0.20, 0.33)	0.62	5.00 (2.98, 8.39)	1.3 × 10^−8^*	3.69 (2.21, 6.14)	5.28 × 10^−5^*
*EXOC6B*	chr2:72754104	C	G	rs61619102	Intron	0.16	0.96 (−0.39, 0.31)	0.84	4.66 (2.79, 7.80)	4.2 × 10^−8^*	4.69 (2.05, 10.75)	2.59 × 10^−4^*

a Using logistic regression model, AD was associated with different SNPs after adjusting for age, sex, years of education, *APOE* ε4, and PCs

b using GWAS GEEPACK (logistic regression utilizing generalized estimating equations), AD was associated with different SNPs, (CD34+CD133+) and SNP:(CD34+CD133+) after adjusting for age, sex, years of education and PCs

c Using Cox proportional hazards regression model, AD incidence was associated with SNP, (CD34+CD133+), the interaction between SNP and (CD34+CD133+) after adjusting for age, sex, years of education, *APOE* ε4, PCs, and vascular diseases

* *P* value significant < 0.05; Chr: chromosome; Pos: position

**Table 6.** The association between circulating CD34+CD133+ cells and the risk of AD in the context of genetic background

rs4144611 (*KIRREL3*)				rs580382 (*KIRREL3*)				rs61619102 (*EXOC6B*)			
Genotype	CD34+CD133+ cutoffs	HR (95% CI)	*P* value	Genotype	CD34+CD133+ cutoffs	HR (95% CI)	*P* value	Genotype	CD34+CD133+ cutoffs	HR (95% CI)	*P* value
TT	25%	0.18 (0.07–0.46)	3.3 × 10^−4^*	CC	25%	0.21 (0.09–0.47)	1.7 × 10^−4^*	CC	25%	0.48 (0.26–0.88)	0.02*
	50%	0.11 (0.03–0.42)	0.001*		50%	0.16 (0.05–0.47)	9.6 × 10^−4^*		50%	0.38 (0.19–0.74)	0.005*
	75%	N/A (zero AD)	0.006^a^*		75%	N/A (zero AD)	0.002^a^*		75%	0.25 (0.08–0.81)	0.02*
GG + TG	25%	1.02 (0.46–2.25)	0.96	TT + CT	25%	1.80 (0.68–4.82)	0.24	GG + GC	25%	2.22 (0.39–12.64)	0.37
	50%	1.54 (0.76–3.15)	0.23		50%	2.44 (1.07–5.56)	0.03*		50%	12.38 (2.00–76.65)	0.007*
	75%	1.39 (0.64–3.05)	0.41		75%	1.80 (0.80–4.07)	0.16		75%	5.68 (1.65–19.61)	0.006*

Participants were first stratified into *KIRREL3* rs4144611 TT *vs.* GG + TG; rs580382 CC *vs.* TT + CT as well as in *EXOC6B* rs61619102 CC *vs.* GG + GC genotype groups. Using Cox proportional hazards regression model, AD incidence was associated with (CD34+CD133+ cutoffs: 25%, 50%, and 75%) after adjusting for age, sex, years of education, *APOE* ε4, and PCs for each genotype group. ^a^ When CD34+CD133+ is higher than the 75% percentile, there are no AD cases among these genotypes, *KIRREL3* rs4144611 TT, *KIRREL3* rs580382 CC, and *EXOC6B*; * *P* value significant < 0.05

## Abbreviations

AD: Alzheimer’s disease
*APOE*: apolipoprotein E
CHD: coronary heart disease
CI: confidence interval
CMB: cerebral microbleeds
DLPFC: dorsolateral prefrontal cortex
EMCs: endothelial mature cells
EPCs: endothelial progenitor cells
*EXOC6B*: exocyst complex component 6B
FHS: Framingham Heart Study
Gen 2: Generation 2
GWAS: genome-wide association study
HR: hazard ratio
HTN: hypertension
*KIRREL3*: kirre like nephrin family adhesion molecule 3
MAF: minor allele frequency
MRI: magnetic resonance imaging
PBMCs: peripheral blood mononuclear cells
PCs: principal components
ROSMAP: Religious Orders Study/Memory and Aging Project
SNPs: single nucleotide polymorphisms
TCV: total cerebral brain volume
WMHI: white matter hyperintensity

## References

[1] Custodia A, Ouro A, Romaus-Sanjurjo D, Pias-Peleteiro JM, de Vries HE, Castillo J, Endothelial progenitor cells and vascular alterations in Alzheimer’s disease. Front Aging Neurosci. 2022;13:811210. 35153724 10.3389/fnagi.2021.811210 PMC8825416

[2] Zhang Z, Gan Q, Han J, Tao Q, Qiu WQ, Madri JA. CD31 as a probable responding and gate-keeping protein of the blood-brain barrier and the risk of Alzheimer’s disease. J Cereb Blood Flow Metab. 2023;43:1027–41. 37051650 10.1177/0271678X231170041 PMC10291450

[3] Vagnucci AH Jr, Li WW. Alzheimer’s disease and angiogenesis. Lancet. 2003;361:605–8. 12598159 10.1016/S0140-6736(03)12521-4

[4] Chou RC, Kane M, Ghimire S, Gautam S, Gui J. Treatment for rheumatoid arthritis and risk of Alzheimer’s disease: a nested case-control analysis. CNS Drugs. 2016;30:1111–20. 27470609 10.1007/s40263-016-0374-z PMC5585782

[5] Drake JD, Chambers AB, Ott BR, Daiello LA; Alzheimer’s Disease Neuroimaging Initiative. Peripheral markers of vascular endothelial dysfunction show independent but additive relationships with brain-based biomarkers in association with functional impairment in Alzheimer’s disease. J Alzheimers Dis. 2021;80:1553–65. 33720880 10.3233/JAD-200759 PMC8150492

[6] Zhang Z, Na H, Gan Q, Tao Q, Alekseyev Y, Hu J, Monomeric C-reactive protein via endothelial CD31 for neurovascular inflammation in an ApoE genotype-dependent pattern: a risk factor for Alzheimer’s disease? Aging Cell. 2021;20:e13501. 34687487 10.1111/acel.13501 PMC8590103

[7] Cao W, Zheng H. Peripheral immune system in aging and Alzheimer’s disease. Mol Neurodegener. 2018;13:51. 30285785 10.1186/s13024-018-0284-2 PMC6169078

[8] Liebner S, Dijkhuizen RM, Reiss Y, Plate KH, Agalliu D, Constantin G. Functional morphology of the blood-brain barrier in health and disease. Acta Neuropathol. 2018;135:311–36. 29411111 10.1007/s00401-018-1815-1 PMC6781630

[9] Rigato M, Avogaro A, Fadini GP. Levels of circulating progenitor cells, cardiovascular outcomes and death: a meta-analysis of prospective observational studies. Circ Res. 2016;118:1930–9. 27073015 10.1161/CIRCRESAHA.116.308366

[10] Yamaguchi T, Kanayasu-Toyoda T, Uchida E. Angiogenic cell therapy for severe ischemic diseases. Biol Pharm Bull. 2013;36:176–81. 23370348 10.1248/bpb.b12-01008

[11] Maslovaric M, Fatic N, Delević E. State of the art of stem cell therapy for ischaemic cardiomyopathy. Part 1. Angiol Sosud Khir. 2019;25:39–52. 31503246 10.33529/ANGIO2019324

[12] Miller-Kasprzak E, Jagodziński PP. Endothelial progenitor cells as a new agent contributing to vascular repair. Arch Immunol Ther Exp (Warsz). 2007;55:247–59. 17659378 10.1007/s00005-007-0027-5

[13] Kong XD, Zhang Y, Liu L, Sun N, Zhang MY, Zhang JN. Endothelial progenitor cells with Alzheimer’s disease. Chin Med J (Engl). 2011;124:901–6. 21518600

[14] Maler JM, Spitzer P, Lewczuk P, Kornhuber J, Herrmann M, Wiltfang J. Decreased circulating CD34^+^ stem cells in early Alzheimer’s disease: evidence for a deficient hematopoietic brain support? Mol Psychiatry. 2006;11:1113–5. 17033629 10.1038/sj.mp.4001913

[15] Kannel WB, Feinleib M, McNamara PM, Garrison RJ, Castelli WP. An investigation of coronary heart disease in families. The Framingham offspring study. Am J Epidemiol. 1979;110:281–90. 474565 10.1093/oxfordjournals.aje.a112813

[16] Cheng S, Cohen KS, Shaw SY, Larson MG, Hwang SJ, McCabe EL, Association of colony-forming units with coronary artery and abdominal aortic calcification. Circulation. 2010;122:1176–82. 20823386 10.1161/CIRCULATIONAHA.109.931279 PMC3050056

[17] Cheng S, Wang N, Larson MG, Palmisano JN, Mitchell GF, Benjamin EJ, Circulating angiogenic cell populations, vascular function, and arterial stiffness. Atherosclerosis. 2012;220:145–50. 22093724 10.1016/j.atherosclerosis.2011.10.015 PMC3277804

[18] Cohen KS, Cheng S, Larson MG, Cupples LA, McCabe EL, Wang YA, Circulating CD34^+^ progenitor cell frequency is associated with clinical and genetic factors. Blood. 2013;121:e50–6. 23287867 10.1182/blood-2012-05-424846 PMC3578962

[19] Muggeridge D, Dodd J, Ross MD. CD34^+^ progenitors are predictive of mortality and are associated with physical activity in cardiovascular disease patients. Atherosclerosis. 2021;333:108–15. 34340831 10.1016/j.atherosclerosis.2021.07.004

[20] Satizabal CL, Beiser AS, Chouraki V, Chêne G, Dufouil C, Seshadri S. Incidence of dementia over three decades in the framingham heart study. N Engl J Med. 2016;374:523–32. 26863354 10.1056/NEJMoa1504327 PMC4943081

[21] DeCarli C, Massaro J, Harvey D, Hald J, Tullberg M, Au R, Measures of brain morphology and infarction in the framingham heart study: establishing what is normal. Neurobiol Aging. 2005;26:491–510. 15653178 10.1016/j.neurobiolaging.2004.05.004

[22] Jefferson AL, Himali JJ, Beiser AS, Au R, Massaro JM, Seshadri S, Cardiac index is associated with brain aging: the Framingham Heart Study. Circulation. 2010;122:690–7. 20679552 10.1161/CIRCULATIONAHA.109.905091 PMC2929763

[23] DeCarli C, Reed T, Miller BL, Wolf PA, Swan GE, Carmelli D. Impact of apolipoprotein E epsilon4 and vascular disease on brain morphology in men from the NHLBI twin study. Stroke. 1999;30:1548–53. 10436099 10.1161/01.str.30.8.1548

[24] Das RR, Seshadri S, Beiser AS, Kelly-Hayes M, Au R, Himali JJ, Prevalence and correlates of silent cerebral infarcts in the Framingham offspring study. Stroke. 2008;39:2929–35. 18583555 10.1161/STROKEAHA.108.516575 PMC2712254

[25] Romero JR, Beiser A, Himali JJ, Shoamanesh A, DeCarli C, Seshadri S. Cerebral microbleeds and risk of incident dementia: the Framingham Heart Study. Neurobiol Aging. 2017;54:94–9. 28347929 10.1016/j.neurobiolaging.2017.02.018 PMC5401784

[26] DeCarli C, Miller BL, Swan GE, Reed T, Wolf PA, Garner J, Predictors of brain morphology for the men of the NHLBI twin study. Stroke. 1999;30:529–36. 10066847 10.1161/01.str.30.3.529

[27] Aljabar P, Heckemann RA, Hammers A, Hajnal JV, Rueckert D. Multi-atlas based segmentation of brain images: atlas selection and its effect on accuracy. Neuroimage. 2009;46:726–38. 19245840 10.1016/j.neuroimage.2009.02.018

[28] Rueckert D, Aljabar P, Heckemann RA, Hajnal JV, Hammers A. Diffeomorphic registration using B-splines. In: Larsen R, Nielsen M, Sporring, editors. Medical image computing and computer-assisted intervention – MICCAI 2006. MICCAI 2006: Proceedings of the 9th International Conference; 2006 Oct 1–6; Copenhagen, Denmark. Berlin: Springer; 2006. pp. 702–9. 10.1007/11866763_86 17354834

[29] Kochunov P, Lancaster JL, Thompson P, Woods R, Mazziotta J, Hardies J, Regional spatial normalization: toward an optimal target. J Comput Assist Tomogr. 2001;25:805–16. 11584245 10.1097/00004728-200109000-00023

[30] Fletcher E, Carmichael O, Decarli C. MRI non-uniformity correction through interleaved bias estimation and B-spline deformation with a template. In: Engineering in medicine and biology society. EMBC 2012: Proceeding of the 2012 Annual International Conference of the IEEE; 2012 Aug 28–Sept 1; San Diego, USA. IEEE; 2012. pp. 106–9. 10.1109/EMBC.2012.6345882 PMC3775836 23365843

[31] Devlin B, Roeder K. Genomic control for association studies. Biometrics. 1999;55:997–1004. 11315092 10.1111/j.0006-341x.1999.00997.x

[32] Boughton AP, Welch RP, Flickinger M, VandeHaar P, Taliun D, Abecasis GR, LocusZoom.js: interactive and embeddable visualization of genetic association study results. Bioinformatics. 2021;37:3017–8. 33734315 10.1093/bioinformatics/btab186 PMC8479674

[33] GTEx Consortium. The Genotype-Tissue Expression (GTEx) project. Nat Genet 2013;45:580–5. 23715323 10.1038/ng.2653 PMC4010069

[34] Ng B, White CC, Klein HU, Sieberts SK, McCabe C, Patrick E, An xQTL map integrates the genetic architecture of the human brain’s transcriptome and epigenome. Nat Neurosci. 2017;20:1418–26. 28869584 10.1038/nn.4632 PMC5785926

[35] Chojdak-Łukasiewicz J, Dziadkowiak E, Zimny A, Paradowski B. Cerebral small vessel disease: a review. Adv Clin Exp Med. 2021;30:349–56. 33768739 10.17219/acem/131216

[36] Kutikhin AG, Sinitsky MY, Yuzhalin AE, Velikanova EA. Shear stress: an essential driver of endothelial progenitor cells. J Mol Cell Cardiol. 2018;118:46–69. 29549046 10.1016/j.yjmcc.2018.03.007

[37] Chen S, Li M, Sun J, Wang D, Weng C, Zeng Y, Human umbilical cord blood-derived CD133^+^CD34^+^ cells protect retinal endothelial cells and ganglion cells in X-irradiated rats through angioprotective and neurotrophic factors. Front Cell Dev Biol. 2022;10:801302. 35223834 10.3389/fcell.2022.801302 PMC8866877

[38] AbuSamra DB, Aleisa FA, Al-Amoodi AS, Jalal Ahmed HM, Chin CJ, Abuelela AF, Not just a marker: CD34 on human hematopoietic stem/progenitor cells dominates vascular selectin binding along with CD44. Blood Adv. 2017;1:2799–816. 29296932 10.1182/bloodadvances.2017004317 PMC5745127

[39] Meregalli M, Farini A, Belicchi M, Torrente Y. CD133^+^ cells isolated from various sources and their role in future clinical perspectives. Expert Opin Biol Ther. 2010;10:1521–8. 20932225 10.1517/14712598.2010.528386

[40] Bigalke B, Schreitmüller B, Sopova K, Paul A, Stransky E, Gawaz M, Adipocytokines and CD34^+^ progenitor cells in Alzheimer’s disease. PLoS One. 2011;6:e20286. 21633502 10.1371/journal.pone.0020286 PMC3102092

[41] Kalaria RN, Kroon SN. Expression of leukocyte antigen CD34 by brain capillaries in Alzheimer’s disease and neurologically normal subjects. Acta Neuropathol. 1992;84:606–12. 1281954 10.1007/BF00227737

[42] Torrente Y, Belicchi M, Sampaolesi M, Pisati F, Meregalli M, D’Antona G, Human circulating AC133^+^ stem cells restore dystrophin expression and ameliorate function in dystrophic skeletal muscle. J Clin Invest. 2004;114:182–95. 15254585 10.1172/JCI20325 PMC449743

[43] Agarwal N, Carare RO. Cerebral vessels: an overview of anatomy, physiology, and role in the drainage of fluids and solutes. Front Neurol. 2021;11:611485. 33519691 10.3389/fneur.2020.611485 PMC7838613

[44] Silva MVF, Loures CMG, Alves LCV, de Souza LC, Borges KBG, Carvalho MDG. Alzheimer’s disease: risk factors and potentially protective measures. J Biomed Sci. 2019;26:33. 31072403 10.1186/s12929-019-0524-y PMC6507104

[45] Untergasser G, Koeck R, Wolf D, Rumpold H, Ott H, Debbage P, CD34^+^/CD133^−^ circulating endothelial precursor cells (CEP): characterization, senescence and *in vivo* application. Exp Gerontol. 2006;41:600–8. 16698211 10.1016/j.exger.2006.03.019

[46] Kapoor A, Gaubert A, Marshall A, Meier IB, Yew B, Ho JK, Increased levels of circulating angiogenic cells and signaling proteins in older adults with cerebral small vessel disease. Front Aging Neurosci. 2021;13:711784. 34650423 10.3389/fnagi.2021.711784 PMC8510558

[47] Huang ZX, Fang J, Zhou CH, Zeng J, Yang D, Liu Z. CD34^+^ cells and endothelial progenitor cell subpopulations are associated with cerebral small vessel disease burden. Biomark Med. 2021;15:191–200. 33496611 10.2217/bmm-2020-0350

[48] Taguchi A, Matsuyama T, Moriwaki H, Hayashi T, Hayashida K, Nagatsuka K, Circulating CD34- positive cells provide an index of cerebrovascular function. Circulation. 2004;109:2972–5. 15184275 10.1161/01.CIR.0000133311.25587.DE

[49] Dalkara T, Alarcon-Martinez L. Cerebral microvascular pericytes and neurogliovascular signaling in health and disease. Brain Res. 2015;1623:3–17. 25862573 10.1016/j.brainres.2015.03.047

[50] Heiss C, Keymel S, Niesler U, Ziemann J, Kelm M, Kalka C. Impaired progenitor cell activity in age-related endothelial dysfunction. J Am Coll Cardiol. 2005;45:1441–8. 15862416 10.1016/j.jacc.2004.12.074

[51] Povsic TJ, Zhou J, Adams SD, Bolognesi MP, Attarian DE, Peterson ED. Aging is not associated with bone marrow-resident progenitor cell depletion. J Gerontol A Biol Sci Med Sci. 2010;65A:1042–50. 10.1093/gerona/glq110 PMC2949333 20591876

[52] Madonna R, Ferdinandy P, De Caterina R, Willerson JT, Marian AJ. Recent developments in cardiovascular stem cells. Circ Res. 2014;115:e71–8. 25477490 10.1161/CIRCRESAHA.114.305567

[53] Al Mheid I, Hayek SS, Ko YA, Akbik F, Li Q, Ghasemzadeh N, Age and human regenerative capacity impact of cardiovascular risk factors. Circ Res. 2016;119:801–9. 27436845 10.1161/CIRCRESAHA.116.308461 PMC5026592

[54] Povsic TJ, Sloane R, Green JB, Zhou J, Pieper CF, Pearson MP, Depletion of circulating progenitor cells precedes overt diabetes: a substudy from the VA enhanced fitness trial. J Diabetes Complications. 2013;27:633–6. 24055327 10.1016/j.jdiacomp.2013.08.004 PMC3874717

[55] Povsic TJ, Sloane R, Pieper CF, Pearson MP, Peterson ED, Cohen HJ, Endothelial progenitor cell levels predict future physical function: an exploratory analysis from the VA enhanced fitness study. J Gerontol A Biol Sci Med Sci. 2016;71:362–9. 26511012 10.1093/gerona/glv180 PMC5013973

[56] Topel ML, Hayek SS, Ko YA, Sandesara PB, Samman Tahhan A, Hesaroieh I, Sex differences in circulating progenitor cells. J Am Heart Assoc. 2017;6:e006245. 28974500 10.1161/JAHA.117.006245 PMC5721840

[57] Tamir-Livne Y, Mubariki R, Bengal E. Adhesion molecule Kirrel3/Neph2 is required for the elongated shape of myocytes during skeletal muscle differentiation. Int J Dev Biol. 2017;61:337–45. 28621431 10.1387/ijdb.170005eb

[58] Martin EA, Muralidhar S, Wang Z, Cervantes DC, Basu R, Taylor MR, Correction: The intellectual disability gene Kirrel3 regulates target-specific mossy fiber synapse development in the hippocampus. Elife. 2016;5:e18706. 27310701 10.7554/eLife.18706 PMC4911213

[59] Ueno H, Sakita-Ishikawa M, Morikawa Y, Nakano T, Kitamura T, Saito M. A stromal cell-derived membrane protein that supports hematopoietic stem cells. Nat Immunol. 2003;4:457–63. 12665856 10.1038/ni916

[60] Mu Khan, Ali I, Jiao W, Wang Y, Masood S, Yousaf MZ, *Ex vivo* expansion of functional human UCB-HSCs/HPCs by coculture with AFT024-*hkirre* cells. Biomed Res Int. 2014;2014:412075. 24719861 10.1155/2014/412075 PMC3955665

[61] Völker LA, Maar BA, Pulido Guevara BA, Bilkei-Gorzo A, Zimmer A, Brönneke H, Neph2/Kirrel3 regulates sensory input, motor coordination, and home-cage activity in rodents. Genes Brain Behav. 2018;17:e12516. 10.1111/gbb.12516 30133126

[62] Taylor MR, Martin EA, Sinnen B, Trilokekar R, Ranza E, Antonarakis SE, Kirrel3-mediated synapse formation is attenuated by disease-associated missense variants. J Neurosci. 2020;40:5376–88. 32503885 10.1523/JNEUROSCI.3058-19.2020 PMC7343328

[63] Baig DN, Yanagawa T, Tabuchi K. Distortion of the normal function of synaptic cell adhesion molecules by genetic variants as a risk for autism spectrum disorders. Brain Res Bull. 2017;129:82–90. 27743928 10.1016/j.brainresbull.2016.10.006

[64] Mao Y, Tu R, Huang Y, Mao D, Yang Z, Lau PK, The exocyst functions in niche cells to promote germline stem cell differentiation by directly controlling EGFR membrane trafficking. Development. 2019;146:dev174615. 31142545 10.1242/dev.174615 PMC6633608

[65] Sun J, Song F, Wang J, Han G, Bai Z, Xie B, Hidden risk genes with high-order intragenic epistasis in Alzheimer’s disease. J Alzheimers Dis. 2014;41:1039–56. 24762948 10.3233/JAD-140054

[66] Hatcher C, Relton CL, Gaunt TR, Richardson TG. Leveraging brain cortex-derived molecular data to elucidate epigenetic and transcriptomic drivers of complex traits and disease. Transl Psychiatry. 2019;9:105. 30820025 10.1038/s41398-019-0437-2 PMC6395652

[67] Martin EA, Woodruff D, Rawson RL, Williams ME. Examining hippocampal mossy fiber synapses by 3D electron microscopy in wildtype and Kirrel3 knockout mice. eNeuro. 2017;4:ENEURO.0088-17.2017. 10.1523/ENEURO.0088-17.2017 PMC5490256 28670619

[68] Prince JE, Brignall AC, Cutforth T, Shen K, Cloutier JF. Kirrel3 is required for the coalescence of vomeronasal sensory neuron axons into glomeruli and for male-male aggression. Development. 2013;140:2398–408. 23637329 10.1242/dev.087262 PMC3653560

[69] Choi SY, Han K, Cutforth T, Chung W, Park H, Lee D, Mice lacking the synaptic adhesion molecule Neph2/Kirrel3 display moderate hyperactivity and defective novel object preference. Front Cell Neurosci. 2015;9:283. 26283919 10.3389/fncel.2015.00283 PMC4517382

[70] Jiang S, Zhang CY, Tang L, Zhao LX, Chen HZ, Qiu Y. Integrated genomic analysis revealed associated genes for Alzheimer’s disease in APOE4 non-carriers. Curr Alzheimer Res. 2019;16:753–63. 31441725 10.2174/1567205016666190823124724

[71] Hisaoka T, Komori T, Kitamura T, Morikawa Y. Abnormal behaviours relevant to neurodevelopmental disorders in Kirrel3-knockout mice. Sci Rep. 2018;8:1408. 29362445 10.1038/s41598-018-19844-7 PMC5780462

[72] Liu YF, Sowell SM, Luo Y, Chaubey A, Cameron RS, Kim HG, Autism and intellectual disability-associated KIRREL3 interacts with neuronal proteins MAP1B and MYO16 with potential roles in neurodevelopment. PLoS One. 2015;10:e0123106. 25902260 10.1371/journal.pone.0123106 PMC4406691

[73] Bhalla K, Luo Y, Buchan T, Beachem MA, Guzauskas GF, Ladd S, Alterations in *CDH15* and *KIRREL3* in patients with mild to severe intellectual disability. Am J Hum Genet. 2008;83:703–13. 19012874 10.1016/j.ajhg.2008.10.020 PMC2668064

[74] Ciaccio C, Leonardi E, Polli R, Murgia A, D’Arrigo S, Granocchio E, A missense *de novo* variant in the *CASK*-interactor *KIRREL3* gene leading to neurodevelopmental disorder with mild cerebellar hypoplasia. Neuropediatrics. 2021;52:484–8. 33853164 10.1055/s-0041-1725964

[75] Anzick S, Thurm A, Burkett S, Velez D, Cho E, Chlebowski C, Chromoanasynthesis as a cause of Jacobsen syndrome. Am J Med Genet A. 2020;182:2533–9. 32841469 10.1002/ajmg.a.61824 PMC11007684

[76] Guerin A, Stavropoulos DJ, Diab Y, Chénier S, Christensen H, Kahr WH, Interstitial deletion of 11q-implicating the *KIRREL3* gene in the neurocognitive delay associated with Jacobsen syndrome. Am J Med Genet A. 2012;158A:2551–6. 22965935 10.1002/ajmg.a.35621

[77] Kalsner L, Twachtman-Bassett J, Tokarski K, Stanley C, Dumont-Mathieu T, Cotney J, Genetic testing including targeted gene panel in a diverse clinical population of children with autism spectrum disorder: findings and implications. Mol Genet Genomic Med. 2018;6:171–85. 29271092 10.1002/mgg3.354 PMC5902398

[78] Evers C, Maas B, Koch KA, Jauch A, Janssen JW, Sutter C, Mosaic deletion of *EXOC6B*: further evidence for an important role of the exocyst complex in the pathogenesis of intellectual disability. Am J Med Genet A. 2014;164:3088–94. 10.1002/ajmg.a.36770 25256811

[79] Frühmesser A, Blake J, Haberlandt E, Baying B, Raeder B, Runz H, Disruption of *EXOC6B* in a patient with developmental delay, epilepsy, and a *de novo* balanced t(2;8) translocation. Eur J Hum Genet. 2013;21:1177–80. 23422942 10.1038/ejhg.2013.18 PMC3778356

[80] Borsani G, Piovani G, Zoppi N, Bertini V, Bini R, Notarangelo L, Cytogenetic and molecular characterization of a *de-novo* t(2p;7p) translocation involving *TNS3* and *EXOC6B* genes in a boy with a complex syndromic phenotype. Eur J Med Genet. 2008;51:292–302. 18424204 10.1016/j.ejmg.2008.02.006

[81] Bjørklund G, Kern JK, Urbina MA, Saad K, El-Houfey AA, Geier DA, Cerebral hypoperfusion in autism spectrum disorder. Acta Neurobiol Exp (Wars), 2018;78:21–9. 29694338

