# Supplementary material for: The association between circulating CD34+CD133+ endothelial progenitor cells and reduced risk of Alzheimer’s disease in the Framingham Heart Study
Source: Explor Med. Author manuscript; Available in PMC 2024 Jun 7. (PMC11160969; doi:10.37349/emed.2024.00216)
Supplement: supplement materials [file NIHMS1996576-supplement-supplement_materials.docx]

**Supplementary Information**

**Supplementary Table 1. Detailed examination of the relationship between number of CD34+CD133+ cells (log transformed) and the risk of AD/dementia risk.**

**Supplementary Table 2. Detailed examination of the relationship between CD34+CD133+ (log transformed) and the risk of diseases by using Cox proportional hazards regression analyses or Body Mass Index (BMI) and white matter hyperintensities (WMHI) by using linear regression analysis.**

**Supplementary Table 3. Examination of the interactions between different progenitor or mature endothelial cells (log transformed) and vascular diseases on the risk of AD/dementia.**

**Supplementary Table 4. Other SNPs from GWAS interaction results of KIRREL3 and EXOC6B*.***

**Supplementary Table 5. The association between CD34+CD133+ and AD incidences after stratification with the genotypes.**

**Supplementary Table 6. Circulating CD34+CD133+ cells for Alzheimer’s disease in the context of genetic background in FHS, stratified by each genotype (additive) adjusting for age, sex, years of education, APOE ɛ4 and PCs.**

**Supplementary Table 7. Comparisons of incident Alzheimer’s disease rates across CD34+CD133+ quartiles in the context of genetic background.**

**Supplementary Figure 1. Flow chart of the study design.**

**Supplementary Figure 2. Q-Q plot of the GWAS analyses.**

**Supplementary Figure 3. The relationships between the *KIRREL3* SNPs, gene expression, and Alzheimer’s disease.**

**Supplementary Figure 4. The relationship between *KIRREL3* expression, SNPs and methylation in the ROSMAP study.**

**Supplementary Figure 5. The relationships between the *EXOC6B* SNPs, gene expression, and Alzheimer’s disease.**

**Supplementary Table 1. Detailed examination of the relationship between number of CD34+CD133+ cells (log transformed) and the risk of AD/dementia risk**

| **CD34+CD133+ cells plus Covariates** | **Alzheimer’s disease** | |  | **Dementia** | |
| --- | --- | --- | --- | --- | --- |
|  | **HR (95% CI)** | **P value** |  | **HR (95% CI)** | **P value** |
| **Model 1: no covariates** | 0.66 (0.47, 0.92)  n = 1597 | **0.02** |  | 0.64 (0.48, 0.85)  n = 1619 | **0.002** |
| **Model 2: Age, sex, years of education** | 0.73 (0.53, 1.03)  n = 1597 | 0.07 |  | 0.70 (0.53, 0.94)  n = 1619 | **0.02** |
| **Model 3: Model 2 + *APOE ɛ4***  **+ vascular diseases** | 0.72 (0.51, 1.03)  n = 1325 | 0.07 |  | 0.68 (0.51, 0.93)  n = 1346 | **0.01** |
| **Model 3 after stratification** |  |  |  |  |  |
| **No vascular diseases^a^** | 1.88 (0.28, 12.75)  n = 251 | 0.52 |  | 1.88 (0.28, 12.75)  n = 251 | 0.52 |
| **Peripheral vascular diseases^b^ only** | 0.68 (0.46, 0.99)  n = 826 | **0.04** |  | 0.66 (0.48, 0.91)  n = 844 | **0.01** |
| **Cerebrovascular diseases^c^ only** | 0.70 (0.44, 1.13)  n = 622 | 0.14 |  | 0.63 (0.42, 0.94)  n = 635 | **0.02** |

Abbreviations: HR = hazard ratio

Cox proportional hazards regression models were used to study the relationship between Log transformed CD34+CD133+ cell numbers per mL and the risk of Alzheimer’s disease (AD) or all-cause dementia after adjusting for the covariates. HR with 95% confidence interval (95% CI) with p values are shown.

Model 1: simple association without confounders

Model 2: Adjusting for age, sex and education

Model 3: Model 2 + *APOE ɛ4* + vascular diseases

a. Model 3 after the stratification for those with no CHD, no HTN, no stroke, no silent infarct, no CMB, and low level of WMHI.

b. Model 3 after the stratification for those with peripheral vascular diseases, e.g., CHD or HTN.

c. Model 3 after the stratification for those with cerebrovascular diseases, e.g., Stroke, silent infarct, CMB or high level of WMHI.

**Supplementary Table 2. Detailed examination of the relationship between CD34+CD133+ (log transformed) and the risk of diseases by using Cox proportional hazards regression analyses or Body Mass Index (BMI) and white matter hyperintensities (WMHI) by using linear regression analysis.**

| **CD34+CD133+ cells plus Covariates** | **Model 1: no covariates** | | **Model 2: Age, sex, education** | | **Model 3: Model 2 + *APOE ɛ4*** | |
| --- | --- | --- | --- | --- | --- | --- |
|  | **HR (95% CI)** | **P value** | **HR (95% CI)** | **P value** | **HR (95% CI)** | **P value** |
| **Peripheral vascular diseases** | 1.10 (0.99-1.21) | 0.07 | 1.15 (1.04-1.27) | **0.0072** | 1.15 (1.03-1.27) | **0.009** |
| **Hypertension** | 1.12 (1.01-1.24) | **0.04** | 1.17 (1.06-1.31) | **0.0032** | 1.17 (1.05-1.30) | **0.0039** |
| **CHD** | 0.88 (0.73-1.05) | 0.15 | 0.92 (0.77-1.11) | 0.40 | 0.91 (0.76-1.10) | 0.34 |
| **Diabetes** | 1.06 (0.83-1.36) | 0.64 | 1.09 (0.85-1.40) | 0.49 | 1.09 (0.85-1.40) | 0.49 |
| **Cerebrovascular diseases** | 1.01 (0.90-1.13) | 0.83 | 1.08 (0.97-1.22) | 0.17 | 1.09 (0.97-1.22) | 0.16 |
| **Stroke** | 1.04 (0.79-1.37) | 0.78 | 1.15 (0.87-1.52) | 0.33 | 1.20 (0.91-1.59) | 0.20 |
| **Silent brain infarcts** | 0.94 (0.74-1.20) | 0.63 | 0.97 (0.76-1.24) | 0.80 | 0.99 (0.77-1.26) | 0.92 |
| **CMB** | 0.97 (0.71-1.34) | 0.86 | 1.06 (0.76-1.49) | 0.72 | 1.10 (0.78-1.54) | 0.59 |
|  | **Beta (SE)** | **P value** | **Beta (SE)** | **P value** | **Beta (SE)** | **P value** |
| **BMI** | 0.024 (0.006) | **0.0002** | 0.020 (0.006) | **0.0019** | 0.018 (0.006) | **0.005** |
| **WMHI** | -0.11 (0.05) | **0.02** | -0.03 (0.04) | 0.43 | -0.03 (0.04) | 0.41 |

Model 1: simple association without confounders; Model 2: Adjusting for age, sex and education; Model 3: Model 2 + *APOE ɛ4*

**Supplementary Table 3. Examination of the interactions between different progenitor or mature endothelial cells (log transformed) and vascular diseases on the risk of AD/dementia**

| **Circulating cells** | **Alzheimer’s disease** | | **All-cause Dementia** | |
| --- | --- | --- | --- | --- |
|  | **HR (95% CI)** | **P value** | **HR (95% CI)** | **P value** |
| **CD34+CD133+** $\boldsymbol{\times}$ **vascular diseases** | 0.48 (0.06, 3.91) | 0.49 | 0.48 (0.09, 2.68) | 0.40 |
| **CD34+CD133-** $\boldsymbol{\times}$ **vascular diseases** | 0.23 (0.02, 3.04) | 0.26 | 0.45 (0.05, 3.91) | 0.47 |
| **CD34-CD133+** $\boldsymbol{\times}$ **vascular diseases** | 72.19 (0.36, 1.45e+04) | 0.11 | 8.96 (0.25, 317.46) | 0.23 |
| **CD34+** $\boldsymbol{\times}$ **vascular diseases** | 0.36 (0.02, 8.26) | 0.52 | 0.39 (0.02, 7.99) | 0.54 |
| **CD34+/KDR+** $\boldsymbol{\times}$ **vascular diseases** | 0.85 (0.14, 5.07) | 0.86 | 0.81 (0.14, 4.71) | 0.82 |
| **CD31+/CD45-** $\boldsymbol{\times}$ **vascular diseases** | 1.45 (0.21, 10.18) | 0.71 | 0.83 (0.21, 3.33) | 0.79 |
| **CD31+** $\boldsymbol{\times}$**vascular diseases** | 0.23 (0.004, 14.47) | 0.48 | 2.05 (0.22, 18.78) | 0.53 |
| **CD31-** $\boldsymbol{\times}$ **vascular diseases** | 13.50 (0.34, 541.95) | 0.17 | 3.88 (0.20, 76.21) | 0.37 |
| **CD31+DIM** $\boldsymbol{\times}$ **vascular diseases** | 0.07 (0.00, 46.63) | 0.42 | 0.06 (0.00, 11.28) | 0.30 |
| **CD31+Lymphoid** $\boldsymbol{\times}$ **vascular diseases** | 540.46 (0.001, 3.80e+08) | 0.36 | 0.29 (0.00, 8.58e+04) | 0.85 |

Cox proportional hazards regression models were used to study the interactions between the proportions (%) of different subtypes of EPCs and EMCs (log transformed) and the vascular diseases on the risk of Alzheimer’s disease (AD) or all-cause dementia after adjusting for age, sex, years of education and APOE ε4. Due to the imbalanced distributions of CD34-CD133- cells, the interactive data analysis on this type of cells was not able to be conducted for the meaningful conclusion. HR with 95% confidence interval (95% CI) for the interactive effects with p values are shown.

**Supplemental Table 4. Other SNPs from GWAS interaction results of *KIRREL3* and *EXOC6B***

| **Gene/Closest gene** | **Chr** | **Position ((GRCh37)** | **Major Allele** | **Minor Allele** | **rs ID** | **Beta** | **se** | **P value** |
| --- | --- | --- | --- | --- | --- | --- | --- | --- |
| ***KIRREL3*** | | | | | | | | |
| *KIRREL3* | 11 | 126436966 | C | T | rs605226 | 1.6199 | 0.2677 | 1.60e-08 |
| *KIRREL3* | 11 | 126436922 | G | T | rs605162 | 1.6169 | 0.2678 | 1.70e-08 |
| *KIRREL3* | 11 | 126437834 | T | A | rs578463 | 1.4686 | 0.2458 | 2.40e-08 |
| *KIRREL3* | 11 | 126437941 | G | A | rs619996 | 1.4646 | 0.2452 | 2.40e-08 |
| *KIRREL3* | 11 | 126439019 | C | T | rs634964 | 1.7946 | 0.3006 | 2.50e-08 |
| ***EXOC6B*** | | | | | | | | |
| *EXOC6B* | 2 | 73082263 | G | A | rs55802296 | 1.4881 | 0.2686 | 2.30e-07 |
| *EXOC6B* | 2 | 73079675 | T | C | rs12614041 | 1.4343 | 0.2594 | 2.44e-07 |
| *EXOC6B* | 2 | 73079965 | T | A | rs56711961 | 1.4348 | 0.2596 | 2.45e-07 |
| *EXOC6B* | 2 | 73079989 | G | C | rs56181835 | 1.4349 | 0.2596 | 2.45e-07 |
| *EXOC6B* | 2 | 73081791 | T | A | rs12615875 | 1.4381 | 0.2602 | 2.45e-07 |
| *EXOC6B* | 2 | 73080639 | G | C | rs2063168 | 1.4556 | 0.2643 | 2.70e-07 |
| *EXOC6B* | 2 | 73076311 | T | C | rs4852897 | 1.4127 | 0.2570 | 2.84e-07 |
| *EXOC6B* | 2 | 73075651 | T | C | rs57264301 | 1.4119 | 0.2569 | 2.86e-07 |
| *EXOC6B* | 2 | 73075644 | A | G | rs59615613 | 1.4114 | 0.2568 | 2.86e-07 |
| *EXOC6B* | 2 | 73075206 | C | T | rs67919884 | 1.4057 | 0.2559 | 2.90e-07 |
| *EXOC6B* | 2 | 73069299 | C | T | rs12619068 | 1.3876 | 0.2530 | 3.04e-07 |
| *EXOC6B* | 2 | 73069973 | G | T | rs1876488 | 1.3875 | 0.2530 | 3.04e-07 |
| *EXOC6B* | 2 | 73068205 | T | A | rs67927720 | 1.3876 | 0.2530 | 3.04e-07 |
| *EXOC6B* | 2 | 73065894 | C | T | rs2135983 | 1.3875 | 0.2530 | 3.04e-07 |
| *EXOC6B* | 2 | 73073494 | A | C | rs4852896 | 1.3855 | 0.2527 | 3.04e-07 |
| *EXOC6B* | 2 | 73072728 | C | T | rs60584321 | 1.3855 | 0.2527 | 3.04e-07 |
| *EXOC6B* | 2 | 73071304 | A | T | rs12614455 | 1.3896 | 0.2534 | 3.04e-07 |
| *EXOC6B* | 2 | 73062631 | G | C | rs11126386 | 1.3878 | 0.2531 | 3.05e-07 |
| *EXOC6B* | 2 | 73064491 | G | A | rs6707107 | 1.3878 | 0.2531 | 3.05e-07 |
| *EXOC6B* | 2 | 73061305 | C | T | rs58448780 | 1.3876 | 0.2531 | 3.07e-07 |
| *EXOC6B* | 2 | 73061116 | G | A | rs4564798 | 1.3876 | 0.2531 | 3.07e-07 |
| *EXOC6B* | 2 | 73060352 | T | G | rs7577528 | 1.3876 | 0.2532 | 3.11e-07 |
| *EXOC6B* | 2 | 73060015 | A | G | rs7586886 | 1.3875 | 0.2532 | 3.11e-07 |
| *EXOC6B* | 2 | 72998061 | T | C | rs6716335 | 1.4199 | 0.2593 | 3.15e-07 |
| *EXOC6B* | 2 | 72998075 | C | T | rs6761253 | 1.4194 | 0.2592 | 3.15e-07 |
| *EXOC6B* | 2 | 73034553 | C | T | rs11126382 | 1.3861 | 0.2537 | 3.34e-07 |
| *EXOC6B* | 2 | 73072819 | A | G | rs112345272 | 1.4133 | 0.2588 | 3.39e-07 |
| *EXOC6B* | 2 | 73030396 | A | G | rs10204141 | 1.3839 | 0.2534 | 3.41e-07 |
| *EXOC6B* | 2 | 73030674 | C | T | rs10496187 | 1.3840 | 0.2535 | 3.42e-07 |
| *EXOC6B* | 2 | 73027972 | A | T | rs12619565 | 1.3835 | 0.2534 | 3.43e-07 |
| *EXOC6B* | 2 | 73013879 | T | A | rs7607226 | 1.3819 | 0.2532 | 3.45e-07 |
| *EXOC6B* | 2 | 73013836 | C | T | rs7593084 | 1.3819 | 0.2532 | 3.45e-07 |
| *EXOC6B* | 2 | 73006888 | A | G | rs4597553 | 1.3897 | 0.2547 | 3.49e-07 |
| *EXOC6B* | 2 | 73003486 | C | T | rs58890506 | 1.3998 | 0.2566 | 3.49e-07 |
| *EXOC6B* | 2 | 72997442 | R | D | NA | 1.4036 | 0.2573 | 3.49e-07 |
| *EXOC6B* | 2 | 73005579 | T | C | rs970577 | 1.3899 | 0.2548 | 3.50e-07 |
| *EXOC6B* | 2 | 73007522 | T | C | rs2068410 | 1.3889 | 0.2546 | 3.50e-07 |
| *EXOC6B* | 2 | 73029379 | A | G | rs7586339 | 1.3936 | 0.2564 | 3.88e-07 |
| *EXOC6B* | 2 | 73082129 | C | T | rs11886503 | 1.4540 | 0.2680 | 4.07e-07 |
| *EXOC6B* | 2 | 73082562 | G | T | rs4852899 | 1.4756 | 0.2731 | 4.51e-07 |
| *EXOC6B* | 2 | 73082563 | C | A | rs4852900 | 1.4760 | 0.2732 | 4.51e-07 |
| *EXOC6B* | 2 | 73023457 | T | C | rs111636941 | 1.3785 | 0.2551 | 4.52e-07 |
| *EXOC6B* | 2 | 73071303 | R | D | NA | 1.4171 | 0.2635 | 5.11e-07 |
| *EXOC6B* | 2 | 73082956 | T | A | rs7567893 | 1.4785 | 0.2785 | 7.12e-07 |

**Supplemental Table 5. The association between CD34+CD133+ and AD incidences after stratification with the genotypes**

| **SNP (Gene)** | **Genotype** | **HR (95% CI)** | **P value** |
| --- | --- | --- | --- |
|  |  |  |  |
|  |  |  |  |
| rs4144611 (***KIRREL3)*** | TT | 0.29 (0.15-0.57) | **4.0e-04** |
|  | GG+TG | 1.13 (0.68-1.89) | 0.64 |
| rs580382 (***KIRREL3)*** | CC | 0.31 (0.17-0.57) | **2.0e-04** |
|  | TT+CT | 1.55 (0.89-2.68) | 0.12 |
| rs61619102 (***EXOC6B)*** | CC | 0.49 (0.31-0.75) | **1.2e-03** |
|  | GG+GC | 2.72 (1.17-6.28) | 0.02 |

After the stratification with each genotype of *KIRREL3* or *EXOC6B* genes in FHS, Cox proportional hazards regression model was used to examine the relationship between CD34+CD133+ EPCs proportion (log transformed) and the AD risk adjusted by age, sex, and years of education, as well as *APOE* ε4 and PCs

**Supplementary Table 6. Circulating CD34+CD133+ cells for Alzheimer’s disease in the context of genetic background in FHS, stratified by each genotype (additive) adjusting for age, sex, years of education, *APOE* ɛ4 and PCs**

| **rs4144611 (*KIRREL3*)** | | | | **rs580382 (*KIRREL3*)** | | | | **rs61619102 (*EXOC6B*)** | | | |
| --- | --- | --- | --- | --- | --- | --- | --- | --- | --- | --- | --- |
| **genotype** | **CD34+CD133+ cutoffs** | **HR**  **(95% CI)** | **P value** | **genotype** | **CD34+CD133+ cutoffs** | **HR**  **(95% CI)** | **P value** | **genotype** | **CD34+CD133+ cutoffs** | **HR**  **(95% CI)** | **P value** |
| **TT**  (N=532,  AD=24) | 25% | 0.18  (0.07-0.46) | **3.3e-04** | **CC**  (N=598,  AD=29) | 25% | 0.21  (0.09-0.47) | **1.7e-04** | **CC**  (N=1023,  AD=44) | 25% | 0.48  (0.26-0.88) | **0.02** |
|  | 50% | 0.11  (0.03-0.42) | **0.001** |  | 50% | 0.16  (0.05-0.47) | **9.6e-04** |  | 50% | 0.38  (0.19-0.74) | **0.005** |
|  | 75% | *N/A*  (Zero AD) | **0.006*** |  | 75% | *N/A*  (Zero AD) | **0.002^a^** |  | 75% | 0.25  (0.08-0.81) | **0.02** |
| **TG**  (N=702,  AD=22) | 25% | 0.71  (0.29-1.75) | 0.46 | **CT**  (N=670,  AD=17) | 25% | 1.35  (0.43-4.23) | 0.60 | **GC**  (N=372,  AD=10) | 25% | 1.50  (0.27-8.45) | 0.64 |
|  | 50% | 0.80  (0.34-1.88) | 0.60 |  | 50% | 1.23  (0.46-3.27) | 0.68 |  | 50% | 7.92  (1.39-45.20) | **0.02** |
|  | 75% | 1.16  (0.43-3.10) | 0.77 |  | 75% | 1.65  (0.60-4.58) | 0.33 |  | 75% | 5.31  (1.39-20.28) | **0.01** |
| **GG**  (N=208,  AD=10) | 25% | 4.17  (0.39-44.56) | 0.24 | **TT**  (N=174,  AD=10) | 25% | 3.82  (0.35-41.05) | 0.27 | **GG^b^**  (N=47,  AD=2) | 25% | 0.00  (0.00-lnf) | NA |
|  | 50% | 20.27  (1.71-240.88) | **0.02** |  | 50% | 25.55  (2.26-288.65) | **0.009** |  | 50% | Infinite  (0.00-lnf) | NA |
|  | 75% | 2.31  (0.53-10.13) | 0.27 |  | 75% | 4.05  (0.84-19.55) | 0.08 |  | 75% | 0.39  (0.00-lnf) | NA |

Cox proportional hazards regression model, AD incidence ~ (CD34+CD133+ cutoffs) + age + sex + years of education + *APOE* ɛ4 + PCs, stratified by genotype.

a. When CD34+CD133+ higher than 75% percentile, there are no AD cases among these genotypes, so log-rank statistics is used instead to compare groups.

b. Not enough subjects to perform the analysis.

**Supplementary Table 7. Comparisons of incident Alzheimer’s disease rates across CD34+CD133+ quartiles in the context of genetic background**

|  |  |  | **Blood CD34+CD133+ Endothelia Progenitor Cells frequency, %** | | | | **P-value^a^** |
| --- | --- | --- | --- | --- | --- | --- | --- |
| **SNP (Gene)** | **Genotype** | **Overall**  **[0.002,0.609]** | **1st Q**  **[0.002,0.020]** | **2nd Q**  **[0.020,0.032]** | **3rd Q**  **[0.032,0.049]** | **4th Q**  **[0.049,0.609]** |  |
|  |  | AD, n (%) | AD, n (%) | AD, n (%) | AD, n (%) | AD, n (%) |  |
| **rs4144611 (*KIRREL3*)** | TT | 35 (6.23%) | 20 (13.25%) | 8 (5.52%) | 7 (4.90%) | 0 (0.00%) | **8.8e-05** |
|  | TG | 30 (4.11%) | 13 (6.95%) | 4 (2.50%) | 6 (3.02%) | 7 (3.83%) | 0.14 |
|  | GG | 14 (6.45%) | 1 (1.64%) | 4 (7.41%) | 5 (10.00%) | 4 (7.69%) | 0.30 |
| **rs580382 (*KIRREL3*)** | CC | 40 (6.36%) | 24 (14.55%) | 8 (5.10%) | 8 (4.85%) | 0 (0.00%) | **2.1e-06** |
|  | CT | 28 (4.01%) | 9 (4.86%) | 5 (3.21%) | 6 (3.24%) | 8 (4.62%) | 0.79 |
|  | TT | 11 (6.11%) | 1 (2.04%) | 3 (6.52%) | 4 (9.52%) | 3 (6.98%) | 0.51 |
| **rs61619102 (*EXOC6B*)** | CC | 59 (5.53%) | 31 (10.23%) | 13 (5.51%) | 10 (3.70%) | 5 (1.94%) | **1.1e-04** |
|  | GC | 18 (4.60%) | 3 (3.57%) | 3 (2.68%) | 7 (6.48%) | 5 (5.75%) | 0.52 |
|  | GG | 2 (4.08%) | 0 (0.00%) | 0 (0.00%) | 1 (7.14%) | 1 (8.33%) | 0.60 |

a. Chi-squared test p-value.

The FHS participants were stratified by genotypes, rs4144611 (*KIRREL3*), rs580382 (*KIRREL3*) or rs61619102 (*EXOC6B*). The AD incident rates across CD34+CD133+ EPC quartiles were compared in each genotype by using Chi-squared test. P values are shown.

**Supplementary Figure 1. Flow chart of the study design**


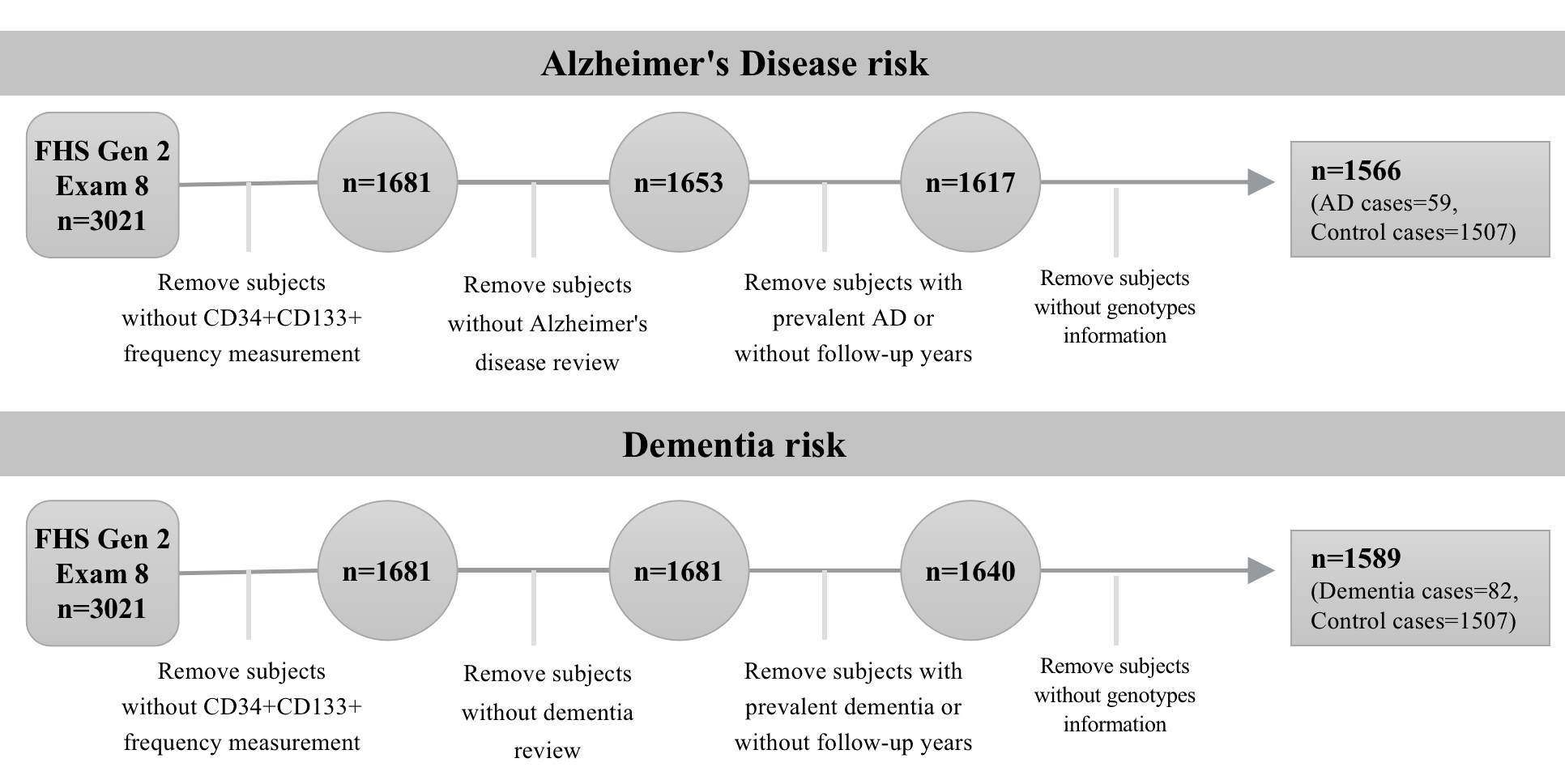


**Supplementary Figure 2. Q-Q plot of the GWAS analyses**


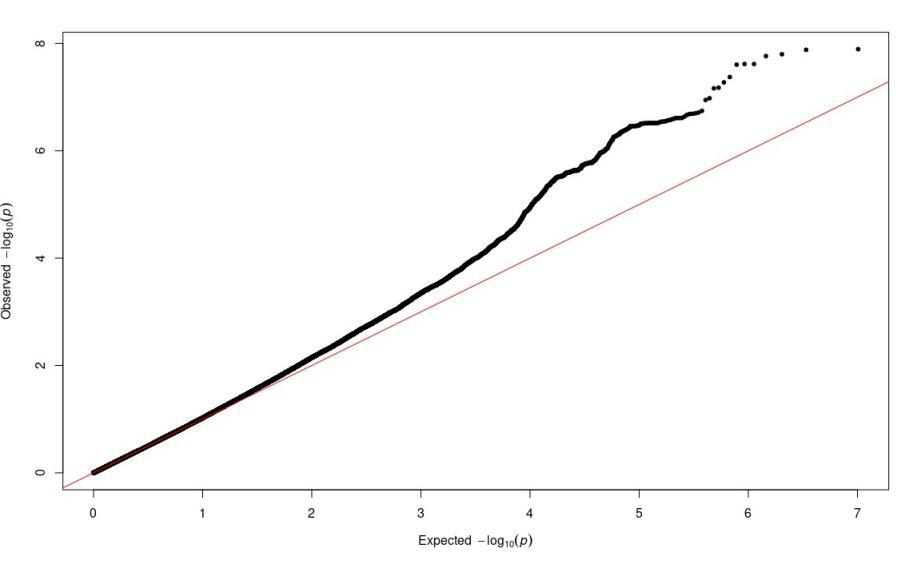


Genomic inflation (λ=1.15 before genomic control)

**Supplementary Figure 3. The relationships between the *KIRREL3* SNPs, gene expression, and Alzheimer’s disease**

We hypothesized that the gene expression levels of KIRREL3 are involved in AD pathology, thus leading to their genotypes having interactive effects with circulating CD34+CD133+ endothelial progenitors for AD risk.

A. by using the ROSMAP dataset and logistic regression, the relationships between KIRREL3 expression in monocytes and brain and AD pathology are shown; B. KIRREL3 mRNA levels across brain regions were compared between AD and controls obtained from Agora; C. interaction between two KIRREL3 SNPs and CD34+CD133+ on AD risk in FHS. ACC = The anterior cingulate cortex; CBE = cerebellum; DLPF = The [dorsolateral prefrontal cortex](https://www.bing.com/ck/a?!&&p=f1f3ba9145ce7482JmltdHM9MTY3MzEzNjAwMCZpZ3VpZD0xNmE2M2Y5NS04MmI1LTZhM2QtMmJmMi0yZmZjODNkMTZiZTMmaW5zaWQ9NTIwNw&ptn=3&hsh=3&fclid=16a63f95-82b5-6a3d-2bf2-2ffc83d16be3&psq=dlpfc+brain&u=a1aHR0cHM6Ly9lbi53aWtpcGVkaWEub3JnL3dpa2kvRG9yc29sYXRlcmFsX3ByZWZyb250YWxfY29ydGV4&ntb=1); FP = The frontal pole; IFG = The inferior frontal gyrus; PCC = The posterior cingulate cortex; PHG = The parahippocampal gyrus; STG = The superior temporal gyrus; TCX = The temporal cortex

**Supplementary Figure 4. The relationship between *KIRREL3* expression, SNPs and methylation in the ROSMAP study**

1. **The relationship between *KIRREL3* expression and the methylation site cg11751545 in the DLPFC region**

**
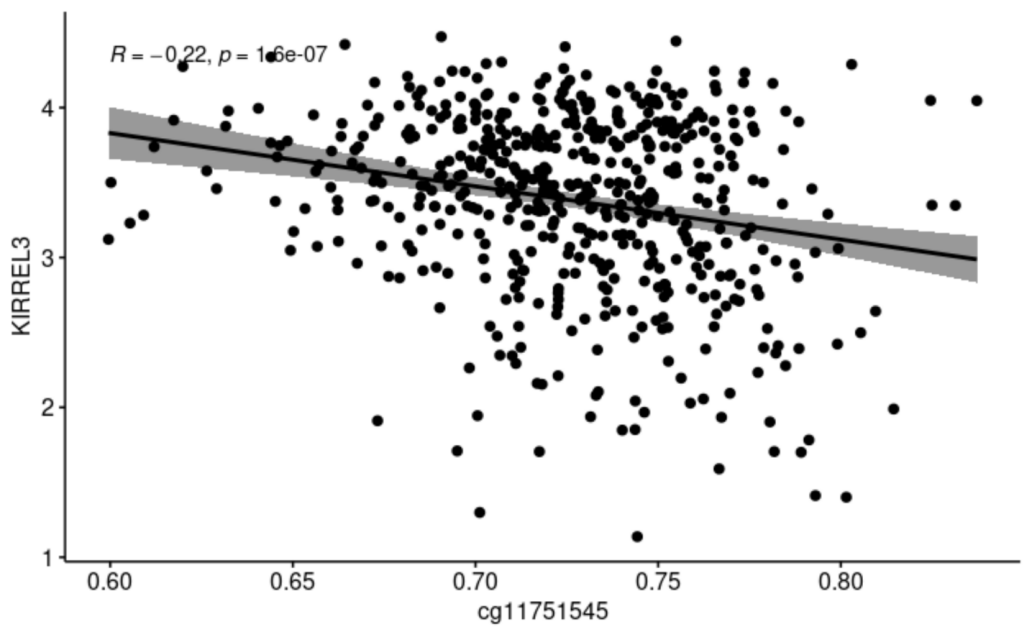
**

1. **The relationships between SNPs and DLPFC methylation in ROSMAP**

**
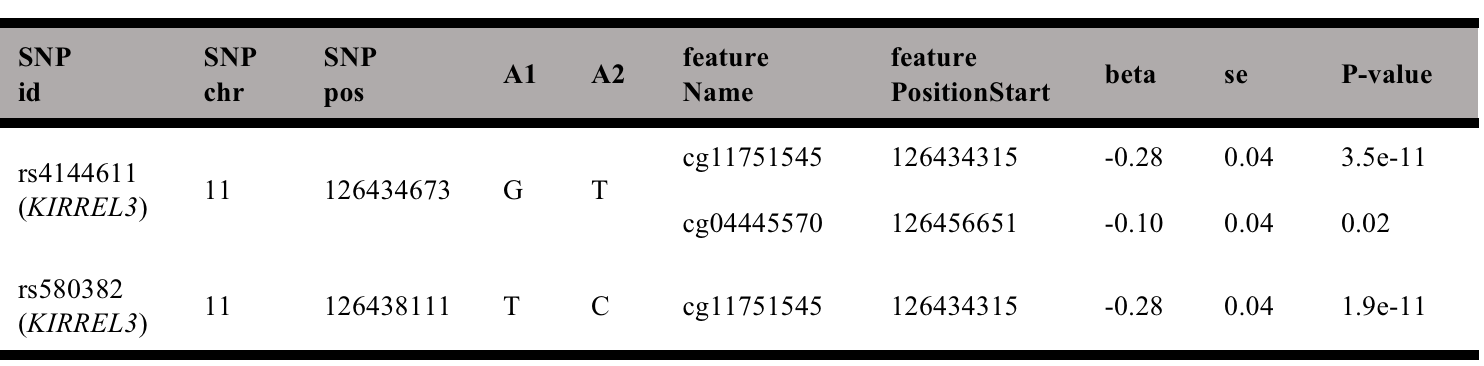
**

**Supplementary Figure 5. The relationships between the *EXOC6B* SNPs, gene expression, and Alzheimer’s disease**

We hypothesized that the gene expression levels of EXOC6B are involved in AD pathology, thus leading to their genotypes having interactive effects with circulating CD34+CD133+ endothelial progenitors for AD risk.

A. by using the ROSMAP dataset and logistic regression, the relationships between EXOC6B expression in monocytes and brain and AD pathology are shown, thus indicating that high peripheral expression of EXOC6B expression was significantly associated with AD risk; B. peripheral eQTL results for the EXOC6B genotype in two types of adipose tissues from GTEx are illustrated, thus suggesting that the EXOC6B cc allele with low peripheral expression and AD risk can be rescued by circulating CD34+CD133+ endothelial progenitor cells for AD risk; C. the interaction between the EXOC6B SNP and CD34+CD133+ cells impacted AD risk in FHS.
